# Posterior cortical atrophy and logopenic variant primary progressive aphasia are more specific for Alzheimer’s disease pathology than probable Alzheimer’s disease

**DOI:** 10.1093/braincomms/fcag304

**Published:** 2026-08-06

**Authors:** Lydia Trudel, Joseph Therriault, Arthur C Macedo, Nesrine Rahmouni, Jean-Paul Soucy, Serge Gauthier, Paolo Vitali, Marie-Christine Guiot, Lili-Naz Hazrati, Pedro Rosa-Neto

**Affiliations:** Translational Neuroimaging Laboratory, Department of Neurology and Neurosurgery, Montreal Neurological Institute, McGill University, Montréal, QC H3A 1A1, Canada; McGill University Research Centre for Studies in Aging, Douglas Hospital Research Centre - Centre intégré universitaire de santé et services sociaux de l’Ouest-de-l’Île-de-Montréal, Verdun, Québec H4H 1R3, Canada; Translational Neuroimaging Laboratory, Department of Neurology and Neurosurgery, Montreal Neurological Institute, McGill University, Montréal, QC H3A 1A1, Canada; McGill University Research Centre for Studies in Aging, Douglas Hospital Research Centre - Centre intégré universitaire de santé et services sociaux de l’Ouest-de-l’Île-de-Montréal, Verdun, Québec H4H 1R3, Canada; Translational Neuroimaging Laboratory, Department of Neurology and Neurosurgery, Montreal Neurological Institute, McGill University, Montréal, QC H3A 1A1, Canada; McGill University Research Centre for Studies in Aging, Douglas Hospital Research Centre - Centre intégré universitaire de santé et services sociaux de l’Ouest-de-l’Île-de-Montréal, Verdun, Québec H4H 1R3, Canada; Translational Neuroimaging Laboratory, Department of Neurology and Neurosurgery, Montreal Neurological Institute, McGill University, Montréal, QC H3A 1A1, Canada; McGill University Research Centre for Studies in Aging, Douglas Hospital Research Centre - Centre intégré universitaire de santé et services sociaux de l’Ouest-de-l’Île-de-Montréal, Verdun, Québec H4H 1R3, Canada; Translational Neuroimaging Laboratory, Department of Neurology and Neurosurgery, Montreal Neurological Institute, McGill University, Montréal, QC H3A 1A1, Canada; PET Unit, McConnell Brain Imaging Centre, Montréal, QC H3A 1A1, Canada; Translational Neuroimaging Laboratory, Department of Neurology and Neurosurgery, Montreal Neurological Institute, McGill University, Montréal, QC H3A 1A1, Canada; McGill University Research Centre for Studies in Aging, Douglas Hospital Research Centre - Centre intégré universitaire de santé et services sociaux de l’Ouest-de-l’Île-de-Montréal, Verdun, Québec H4H 1R3, Canada; Translational Neuroimaging Laboratory, Department of Neurology and Neurosurgery, Montreal Neurological Institute, McGill University, Montréal, QC H3A 1A1, Canada; McGill University Research Centre for Studies in Aging, Douglas Hospital Research Centre - Centre intégré universitaire de santé et services sociaux de l’Ouest-de-l’Île-de-Montréal, Verdun, Québec H4H 1R3, Canada; Department of Neuropathology, Montreal Neurological Institute, McGill University, Montreal, QC H3A 1A1, Canada; Department of Neuropathology, Montreal Neurological Institute, McGill University, Montreal, QC H3A 1A1, Canada; Translational Neuroimaging Laboratory, Department of Neurology and Neurosurgery, Montreal Neurological Institute, McGill University, Montréal, QC H3A 1A1, Canada; McGill University Research Centre for Studies in Aging, Douglas Hospital Research Centre - Centre intégré universitaire de santé et services sociaux de l’Ouest-de-l’Île-de-Montréal, Verdun, Québec H4H 1R3, Canada; Peter O’Donnell Jr. Brain Institute (OBI), University of Texas Southwestern Medical Centre (UTSW), Dallas, TX 75390, USA

**Keywords:** posterior cortical atrophy (PCA), logopenic primary progressive aphasia (lvPPA), Alzheimer’s disease neuropathologic change (ADNC), clinical-pathological correlation, atypical Alzheimer’s disease

## Abstract

Accurately diagnosing Alzheimer’s disease is challenging, as 15–30% of individuals diagnosed clinically lack evidence of Alzheimer’s disease neuropathological changes (ADNC) at autopsy. While the typical amnestic presentation of Alzheimer’s disease may have limited specificity due to coexisting age-related pathologies, atypical presentations such as logopenic variant primary progressive aphasia (lvPPA) or posterior cortical atrophy (PCA) are often associated with ADNC. This study evaluated how well clinical diagnoses of amnestic and atypical Alzheimer’s disease predict ADNC and how co-pathologies contribute to clinical-pathological discordance. To evaluate how clinical diagnosis predicts ADNC, we calculated positive predictive values (PPVs) and negative predictive values (NPVs) for clinical diagnoses of PCA, lvPPA, amnestic Alzheimer’s disease or non-Alzheimer’s disease dementia for ADNC, using four neuropathological thresholds defined by combinations of the Consortium to Establish a Registry for Alzheimer’s disease (CERAD) neuritic plaque frequency (moderate or frequent) and Braak stage (III–VI or V–VI). Analyses were replicated *in vivo* using amyloid- and tau-PET positivity (A+T+) as a surrogate for Alzheimer’s disease pathology. We also quantified the frequency of co-pathologies and assessed their contribution to false-positive Alzheimer’s disease diagnoses. This study included 6363 autopsy-confirmed cases from the National Alzheimer’s Coordinating Center (NACC) database (mean age: 80.0 ± 11.4 years; 46.0% female) and 76 participants from the Translational Biomarkers in Aging and Dementia (TRIAD) cohort (mean age: 67.9 ± 9.94 years; 55.3% female). The PPV of probable Alzheimer’s disease dementia ranged from 58 to 80%, while PCA and lvPPA showed higher PPVs (81–96% and 80–92%, respectively) for ADNC. The NPV of probable Alzheimer’s disease dementia ranged from 66 to 83%, compared with 38–58% for PCA and lvPPA. In TRIAD, individuals with PCA or lvPPA had 100% PPV for Alzheimer’s disease biomarker abnormality, whereas amnestic Alzheimer’s disease dementia showed a PPV of 78.1%. Among Alzheimer’s disease dementia cases, 20.2% were ADNC-negative, with a higher frequency of false-positive diagnoses in late-onset compared with early-onset cases (23.8% versus 6.9%). Among ADNC-negative Alzheimer’s disease dementia cases, vascular pathology was the most common pathology (early-onset: 93%; late-onset: 98%). In the late-onset group, limbic-predominant age-related TDP-43 encephalopathy occurred in 31% of the cases, followed by Lewy body pathology (28.8%) and primary age-related tauopathy (24.3%). Visuospatial and language variants of clinical Alzheimer’s disease offer excellent predictive value for underlying Alzheimer’s disease pathology, both *in vivo* and at autopsy, outperforming the amnestic presentation classically associated with Alzheimer’s disease. Clinical-pathological discordance in amnestic Alzheimer’s disease dementia is frequently associated with coexisting age-related and vascular pathologies, highlighting the complexity of interpreting clinical Alzheimer’s disease diagnoses in the absence of ADNC.

## Teaser

Trudel *et al*. report that atypical Alzheimer’s disease syndromes are highly specific indicators of autopsy-confirmed Alzheimer’s disease pathology. By contrast, clinically diagnosed amnestic Alzheimer’s disease dementia is more heterogeneous and in a substantial proportion of cases reflects non-Alzheimer’s disease pathologies, including Lewy body disease, vascular disease and limbic-predominant age-related TDP-43 encephalopathy.

## Graphical Abstract

**Graphical Abstract:**
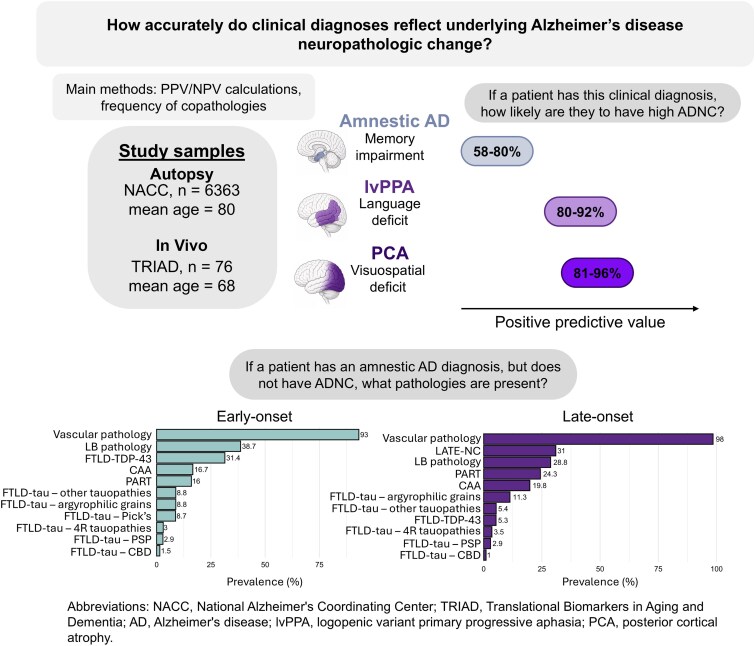

## Introduction

The diagnosis of Alzheimer’s disease based on clinical criteria is challenging, with 15–30% of individuals diagnosed with Alzheimer's disease having insufficient evidence of Alzheimer's disease neuropathological changes (ADNC) at autopsy^1^ or by PET imaging assessments.^2^ In particular, the amnestic syndrome that characterizes the most common clinical presentation of Alzheimer's disease is frequently associated with other common age-related pathological processes affecting the medial temporal lobe, such as primary age-related tauopathy (PART)^3^ and limbic-predominant age-related TDP-43 encephalopathy (LATE).^4^ Therefore, the amnestic-predominant clinical syndrome may have limited specificity for ADNC.

Although ADNC are most commonly associated with an amnestic clinical presentation, they can manifest across a broader range of non-amnestic syndromes with varying degrees of pathological specificity. At one end of this range, corticobasal syndrome (CBS) and behavioural variant frontotemporal dementia are only rarely associated with underlying ADNC^5-9^ and are more commonly driven by non-AD pathologies. In contrast, logopenic variant primary progressive aphasia (lvPPA) and posterior cortical atrophy (PCA) represent atypical clinical phenotypes with much stronger and more consistent association with ADNC despite their non-amnestic clinical presentation.^10-12^ Accordingly, these two phenotypes provide a particularly informative context in which to examine the specificity of clinical expression for ADNC and to determine whether the burden and profile of co-pathologies differ from those observed in amnestic-predominant AD.

Here, in 6363 individuals with neuropathological assessments and 76 individuals assessed *in vivo*, we evaluated how well clinical diagnoses of amnestic and atypical Alzheimer's disease predict ADNC and how the presence of co-pathologies contributes to misdiagnoses. We hypothesized that, despite their atypical clinical presentation, lvPPA and PCA would demonstrate greater pathological specificity for ADNC than the classical amnestic presentation.

## Materials and methods

### Participants

#### Neuropathological cohort

We included 6363 participants from the National Alzheimer’s Coordinating Center (NACC) database (mean age: 80.0 ± 11.4 years; 46.0% female) who had both clinical assessments during life and neuropathological evaluation at autopsy. The NACC compiles standardized clinical and neuropathological information from individuals enrolled at Alzheimer’s Disease Research Centers (ADRCs) across the USA. Clinical data were obtained from the NACC Uniform Dataset (UDS) and neuropathological data from the Neuropathology (NP) dataset, using the December 2024 data freeze. These data were contributed by ADRCs funded by the National Institute of Health/National Institute on Aging.^13^ In total, 39 ADRCs contributed data to this study. All participants provided informed consent for clinical assessments and autopsy procedures, following protocols approved by the institutional review boards at each ADRC. Demographic characteristics of the NACC sample can be found in Table 1.

**Table 1:** Demographic characteristics of the NACC sample

	Probable Alzheimer's disease dementia *N* = 4557	lvPPA *N* = 59	PCA *N* = 53	Non-AD dementia *N* = 1694	All *N* = 6363
Age, mean (SD), years	82.1 (10.8)	74.1 (8.14)	70.1 (8.54)	74.8 (11.2)	80.0 (11.4)
Female, no. (%)	2262 (49.6)	26 (44.1)	25 (47.2)	613 (36.2)	2926 (46.0)
CDR-SB, mean (SD)	11.5 (5.74)	11.7 (5.96)	11.8 (5.00)	11.3 (5.79)	11.4 (5.75)
Education, mean (SD), years	16.0 (8.61)	18.1 (11.0)	19.1 (16.2)	17.0 (10.9)	16.4 (9.38)
APOE ɛ4 status, no. (%)					
Non-carriers	1829 (40.1)	31 (52.5)	29 (54.7)	956 (56.4)	2845 (44.7)
Heterozygotes	1773 (38.9)	19 (32.2)	18 (34.0)	448 (26.4)	2258 (35.5)
Homozygotes	486 (10.7)	5 (8.5)	5 (9.4)	64 (3.8)	560 (8.8)
Missing	469 (10.3)	4 (6.8)	1 (1.9)	226 (13.3)	700 (11.0)

#### *In vivo* cohort

We included 76 individuals diagnosed with either probable Alzheimer's disease dementia, lvPPA or PCA enrolled in the Translational Biomarkers in Aging and Dementia (TRIAD) cohort (mean age: 67.9 ± 9.94 years; 55.3% female). TRIAD, initiated in 2017 at the McGill Centre for Studies in Aging, is an ongoing longitudinal study that recruits participants across the cognitive spectrum, ranging from unimpaired individuals to those with mild cognitive impairment (MCI) or dementia. Detailed eligibility criteria have been reported previously.^14^ General exclusion criteria included recent or current cancer, severe psychiatric illness, history of traumatic brain injury with loss of consciousness, recent or ongoing substance or alcohol abuse and contraindications to MRI or PET procedures. For this analysis, only participants who underwent both amyloid- and tau-PET imaging, along with comprehensive clinical assessments, were included. The TRIAD protocol was approved by the Montreal Neurological Institute PET working committee and the Research Ethics Board of the Douglas Mental Health University Institute. Written informed consent was obtained from all participants or, when applicable, their caregivers. Demographic characteristics of the TRIAD sample can be found in Table 2.

**Table 2:** Demographic characteristics of the TRIAD sample

	AD dementia *N* = 32	lvPPA *N* = 16	PCA *N* = 28	All *N* = 76
Age, mean (SD), years	68.4 (11.4)	72.9 (8.75)	64.5 (7.48)	67.9 (9.94)
Female, no. (%)	14 (43.8%)	10 (62.5%)	18 (64.3%)	42 (55.3%)
MMSE, mean (SD)	20.5 (6.19)	22.4 (5.72)	18.8 (6.56)	20.2 (6.30)
Missing	1 (3.1%)	1 (6.3%)	0 (0%)	2 (2.6%)
Education, mean (SD), years	13.5 (4.49)	14.4 (4.56)	15.9 (2.74)	14.6 (4.03)
Missing	1 (3.1%)	0 (0%)	0 (0%)	1 (1.3%)
APOE ɛ4 status, no. (%)				
Non-carriers	14 (43.8%)	6 (37.5%)	7 (25.0%)	27 (35.5%)
Homozygotes	12 (37.5%)	4 (25.0%)	9 (32.1%)	25 (32.9%)
Heterozygotes	6 (18.8%)	0 (0%)	3 (10.7%)	9 (11.8%)
Missing	0 (0%)	6 (37.5%)	9 (32.1%)	15 (19.7%)

### Clinical assessments

#### Neuropathological sample (National Alzheimer’s Coordinating Center)

All cases included in this study had neuropathological information collected using the NACC Neuropathology Form version 10 or later, ensuring consistency across centres. Clinical variables were obtained from the UDS, which provides demographic data (age, sex, education), cognitive test results and clinical diagnoses based on standardized criteria.^15^

Clinical diagnosis in the UDS is assigned at each centre by an experienced clinician or consensus panel, based on history, neurological examination and cognitive testing. Diagnoses are coded into standardized categories (normal cognition, MCI, Alzheimer's disease dementia and non-AD dementias). Probable and possible Alzheimer's disease dementia are defined according to the 2011 NINCDS-ADRDA criteria,^16^ which require an amnestic presentation with progressive memory impairment and impairment in at least one other cognitive domain. Lewy body dementia and frontotemporal lobar degeneration (FTLD) syndromes are diagnosed according to consensus clinical criteria.^17,18^ Primary progressive aphasia (PPA) is subtyped following consensus criteria into logopenic, semantic or non-fluent/agrammatic variants^11^; cases meeting criteria for the lvPPA are considered to reflect an atypical clinical presentation of AD. PCA is also recorded and defined according to consensus guidelines,^10^ characterized by predominant visuospatial impairment.

For this study, we used the primary clinical diagnosis variable from the most recent UDS visit prior to death to define each participant’s final clinical classification, including probable Alzheimer's disease dementia, lvPPA, PCA and other non-AD dementias. The non-AD dementia group included behavioural variant FTD (*n* = 538), FTD-not otherwise specified (*n* = 42), FTLD with motor disorder (*n* = 21), dementia with Lewy bodies (*n* = 573), non-fluent/agrammatic variant PPA (*n* = 124), semantic variant PPA (*n* = 115), PPA not otherwise specified (*n* = 71) and vascular dementia (*n* = 210).

#### Translational Biomarkers in Aging and Dementia

All participants underwent evaluation at the McGill Center for Studies in Aging, a specialized memory clinic focused on the diagnosis and management of neurodegenerative disorders. Participant evaluations consisted of a review of medical history, an interview with both the participant and their study partner, a neurologic examination conducted by a dementia specialist and a comprehensive neuropsychological assessment. Participants were classified as cognitively unimpaired (CU; not meeting criteria for MCI or dementia),^19^ as having single- or multidomain amnestic MCI^20^ or as having either amnestic or atypical Alzheimer's disease according to established diagnostic criteria.^10,11,16^ Atypical Alzheimer's disease syndromes were diagnosed based on established clinical criteria emphasizing the core language profile, neuropsychological performance and supporting clinical features and were determined by multidisciplinary consensus. Clinical diagnoses were established without knowledge of PET imaging results (i.e. patients received a clinical diagnosis prior to any comparison with biomarker-defined AD). Clinical diagnosis and PET imaging were performed within the same study visit (typically within 3 months), minimizing the potential impact of phenotypic evolution between diagnosis and biomarker assessment.

### Neuropathological assessments

For each participant, we used the last available clinical evaluation prior to death to maximize temporal proximity to autopsy. On average, the interval between the last visit and death was 22 months. Neuropathological information was derived from the NACC Neuropathology Dataset, which relies on standardized assessments based on the NIA–Alzheimer’s Association (NIA-AA) criteria.^21^ Tissue sampling was performed systematically in selected brain regions, including the hippocampus, frontal, temporal and parietal cortices, as well as the brainstem. Histopathological evaluation included amyloid-β (Aβ) plaque distribution, neurofibrillary tangle pathology with Braak staging and neuritic plaque density according to CERAD criteria.^21^ We defined four ADNC thresholds: (i) Braak III–IV with CERAD 2, (ii) Braak III–IV with CERAD 3, (iii) Braak V–VI with CERAD 2 and (iv) Braak V–VI with CERAD 3.^1^ Additional assessments covered FTLD with TDP-43 inclusions (FTLD-TDP-43) and with tau pathology (FTLD-tau, encompassing both 3R and 4R tau),^22^ cerebrovascular pathology,^23^ Lewy body (LB) pathology^24^ and LATE-NC.^4^ The presence of cerebral amyloid angiopathy (CAA) was also assessed using amyloid stains (e.g. Congo red, thioflavin-S or Aβ immunostaining). PART was defined by Thal or CERAD scores of 0 with Braak stages I–IV.^3,25^ Vascular neuropathology was operationalized using the NACCVASC variable from the NP dataset, which captures the presence of one or more ischaemic, haemorrhagic or other vascular brain lesions (e.g. infarcts, haemorrhages and related vascular pathology) assessed at autopsy.

### Neuroimaging

In the TRIAD cohort, procedures for amyloid- and tau-PET imaging have been detailed elsewhere.^26,27^ Briefly, participants underwent [^18^F]AZD4694 and [^18^F]MK6240 PET scans on a Siemens High Resolution Research Tomograph (HRRT) dedicated to brain imaging. Standardized uptake value ratio (SUVR) images for [^18^F]AZD4694 were generated using the whole cerebellum as the reference region, while [^18^F]MK6240 SUVRs were normalized to the inferior cerebellar grey matter. A global amyloid-PET SUVR was calculated by averaging values across precuneus, prefrontal, orbitofrontal, parietal, temporal and cingulate cortices. Individuals with an [^18^F]AZD4694 SUVR greater than 1.55 were classified as amyloid-positive, consistent with a threshold previously validated by our group through complementary methods, including CSF Aβ42/40 ratios, Gaussian mixture modelling and ROC analyses.^26^ Tau-PET positivity was defined using a meta-ROI SUVR cut-off of 1.24, as established in prior work based on a reference group of amyloid-negative, CU young adults (<25 years of age).^2^

### Statistical analysis

Analyses were performed in R version 2024.04.2, and *P*-values were considered significant at 0.05. For analyses in the autopsy dataset, we started by calculating, for each ADNC threshold, the positive predictive value (PPV) and negative predictive value (NPV) for clinical groups diagnosed as probable AD, PCA or lvPPA. PPV was defined as the proportion of individuals clinically classified as a given clinical phenotype who met neuropathologic criteria for AD, whereas NPV was defined as the proportion of individuals not clinically classified as a given clinical phenotype who did not meet neuropathologic criteria for AD. Differences in PPV and NPV across clinical groups were evaluated using *χ*² tests for equality of proportions, followed by pairwise comparisons with *P*-values adjusted using the false discovery rate (FDR) to account for multiple comparisons. We defined the benchmark PPV as the PPV of all-cause dementia for ADNC, representing the baseline probability that a person with dementia has ADNC, against which the PPV of other diagnoses for ADNC could be compared. Sensitivity analyses calculating the PPV in early- versus late-onset of symptom subsets were performed (early-onset *N* = 1968; late-onset *N* = 4291; missing *N* = 104). Then, we calculated the proportion of participants with exhibiting different pathologies across clinical diagnoses, with sensitivity analyses performed in early- versus late-onset subsets. For several neuropathological measures, data were not available for all participants. Consequently, the proportion of individuals positive for each pathology was calculated using only cases with available data, thereby providing accurate estimates while accounting for missing values. Differences in pathology frequency between clinical groups (AD dementia versus lvPPA or PCA) were assessed using two-sample proportion tests based on the number of participants positive for each pathology and the total number in each group, with *P*-values adjusted for multiple comparisons using the FDR method. We then assessed the clinical-pathological discordance in individuals with a clinical diagnosis of AD. To do so, Alzheimer's disease neuropathologic status was classified as ADNC-positive (intermediate/high Alzheimer's disease neuropathologic change) or ADNC-negative, and differences in the proportion of ADNC-negative cases with a clinical diagnosis of Alzheimer's disease (i.e. false positives) between onset groups were evaluated using a *χ*² test. To visualize co-occurrence patterns among neuropathological features in false-positive Alzheimer's disease diagnoses, we generated UpSet plots using the UpSetR package in R. All pathology variables were coded as binary (1 = present, 0 = absent). For single pathologies, counts were defined as the number of participants with that pathology present and all other pathologies equal to 0 or missing, while the denominator included all participants with observed data for the target pathology, regardless of missing data in other pathologies. For combinations of two or more pathologies, counts were defined as the number of participants with all pathologies in the combination present, while other pathologies could be 0 or missing; the denominator included only participants with observed data for all pathologies in the combination. Percentages were then calculated as the count divided by the corresponding denominator, reflecting the proportion of participants with the observed pathology or combination. We additionally computed marginal frequency estimates for each individual pathology to summarize the overall distribution of individual pathologies across the cohort. Specifically, for each marker, we calculated the proportion of cases in which the pathology was present among all individuals with available (non-missing) assessments for that marker. This approach provides a global estimate of pathology burden independent of co-occurring conditions, allowing for interpretation of the overall frequency of each pathology irrespective of combination structure. Finally, we quantified the association between individual pathologies and false-positive Alzheimer's disease dementia diagnoses, in both early-and late-onset groups, using a logistic regression model to calculate odds ratios (ORs) with 95% confidence intervals (CIs), adjusting for age and sex. PART was not included in these models due to strong inflation of effect estimates arising from structural overlap with the neuropathological criteria used to define ADNC status.

For the *in vivo* analyses, PPV was calculated for each clinical group (AD dementia, PCA, lvPPA) as the proportion of individuals within that group who exhibited the specified biomarker profile for Alzheimer's disease proteinopathy (i.e. A+T+). NPV was calculated as the proportion of individuals within each clinical group who did not exhibit the A+T+ biomarker profile. PPV could not be calculated for lvPPA and PCA in the A+T− group, as no cases with this biomarker profile were observed. Differences in PPV and NPV across clinical groups were assessed using a *χ*² test of proportions; when any expected cell counts were below five, Fisher’s exact test was applied. Pairwise comparisons between clinical groups were conducted using Fisher’s exact test with FDR correction for multiple testing.

## Results

### Positive predictive value of clinical diagnoses for different histopathological severity

**Figure 1:**
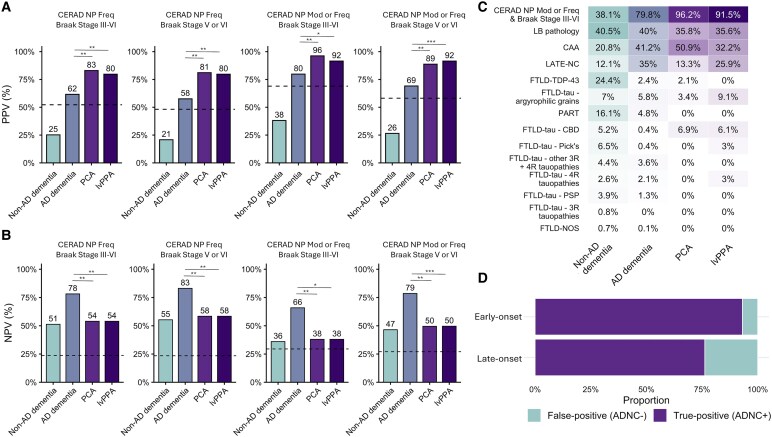
**PPV of clinical diagnoses and co-pathology frequency across neuropathological thresholds.** (**A**) PPV for each clinical diagnosis group (amnestic AD, PCA, lvPPA and non-AD dementia; *N* = 6363), stratified by neuropathological threshold. Thresholds are defined by combinations of CERAD neuritic plaque frequency (moderate or frequent) and Braak stage (III–VI or V–VI). Differences in PPV across clinical groups were evaluated using *χ*² tests for equality of proportions, followed by pairwise comparisons with *P*-values adjusted using the FDR correction. Significance is denoted as *P* < 0.05 (*), *P* < 0.01 (**) and *P* < 0.001 (***). (**B**) NPV for each clinical diagnosis group (amnestic AD, PCA, lvPPA and non-AD dementia; *N* = 6363), stratified by neuropathological threshold. Thresholds are defined by combinations of CERAD neuritic plaque frequency (moderate or frequent) and Braak stage (III–VI or V–VI). Differences in NPV across clinical groups were evaluated using *χ*² tests for equality of proportions, followed by pairwise comparisons with *P*-values adjusted using the FDR correction. Significance is denoted as *P* < 0.05 (*), *P* < 0.01 (**) and *P* < 0.001 (***). (**C**) Percentage of individuals exhibiting different neuropathological findings in clinical groups diagnosed with non-AD dementia, amnestic Alzheimer's disease dementia, PCA and lvPPA. (**D**) Proportion among individuals with Alzheimer's disease dementia (N = 4557), with and without ADNC, in early- and late-onset groups. AD, Alzheimer’s disease; ADNC, Alzheimer’s disease neuropathologic changes; CAA, cerebral amyloid angiopathy; CBD, corticobasal degeneration; CERAD NP, Consortium to Establish a Registry for Alzheimer’s Disease neuritic plaque; Freq, frequent; FTLD-tau, frontotemporal lobar degeneration with tau pathology; FTLD-TDP-43, frontotemporal lobar degeneration with TDP-43 pathology; LB, Lewy body; LATE-NC, limbic-predominant age-related TDP-43 encephalopathy neuropathologic change; lvPPA, logopenic variant primary progressive aphasia; Mod, moderate; NOS, not otherwise specified; NPV, negative predictive value; PCA, posterior cortical atrophy; PART, primary age-related tauopathy; PSP, progressive supranuclear palsy; PPV, positive predictive value; 3R, three-repeat tau; 4R, four-repeat tau.

We calculated the PPV across diagnoses (non-AD dementia, clinical Alzheimer's disease dementia, lvPPA or PCA), stratified by histopathological severity (Fig. 1A). PPV estimates and 95% CIs are provided in Supplementary Table 1. The PPV for the clinical diagnosis of probable Alzheimer's disease dementia ranged from 58 to 80%, with the best result achieved when the neuropathological diagnosis was moderate or frequent neuritic plaques with Braak stages III–VI. The PPV for the clinical diagnosis of PCA ranged from 81 to 96%, with the best result achieved when the neuropathological diagnosis was moderate or frequent neuritic plaques with Braak stages III–VI. The PPV for the clinical diagnosis of lvPPA ranged from 80 to 92%, with the best result achieved when the neuropathological diagnosis was moderate or frequent plaques with Braak stages from either III–VI or V–VI. *χ*² tests for equality of proportions consistently showed a difference between atypical presentations of Alzheimer's disease and the amnestic Alzheimer's disease syndrome. *P*-values are listed in Supplementary Table 2.

We calculated benchmark PPVs for each ADNC threshold (frequent neuritic plaques with Braak V–VI: 0.482; moderate to frequent neuritic plaques with Braak V–VI: 0.581; frequent neuritic plaques with Braak III–VI: 0.522; moderate to frequent neuritic plaques with Braak III–VI: 0.690). When comparing the PPV with the benchmark PPVs, we found that the PPV for clinical Alzheimer's disease dementia was consistently between 10 and 12% higher than the benchmark PPV, while lvPPA and PCA were roughly 28–34% higher across all histopathological thresholds (Supplementary Fig. 1A).

### Negative predictive value of clinical diagnoses for different histopathological severity

We calculated the NPV across diagnoses stratified by histopathological severity (Fig. 1B). NPV estimates and 95% CIs are provided in Supplementary Table 3. The NPV for the clinical diagnosis of probable Alzheimer's disease dementia ranged from 66 to 83%, with the best result achieved when the neuropathological diagnosis was frequent neuritic plaques with Braak stages V or VI. The NPV for the clinical diagnosis of both lvPPA PCA ranged from 38 to 58%, with the best result achieved when the neuropathological diagnosis was frequent neuritic plaques with Braak stages V or VI. *χ*² tests for equality of proportions consistently showed a difference between atypical presentations of Alzheimer's disease and the amnestic Alzheimer's disease syndrome. *P*-values are listed in Supplementary Table 4.

We calculated benchmark NPVs for each ADNC threshold (frequent neuritic plaques with Braak V–VI: 0.235; moderate to frequent neuritic plaques with Braak V–VI: 0.271; frequent neuritic plaques with Braak III–VI: 0.237; moderate to frequent neuritic plaques with Braak III–VI: 0.295). When comparing the NPV with the benchmark NPVs, we found that the NPV for clinical Alzheimer's disease dementia was between 36 and 60% higher than the benchmark NPV, while lvPPA and PCA were between 8.5 and 35% higher, with smallest difference with benchmark for the moderate or frequent neuritic plaques with Braak stages III–VI threshold (Supplementary Fig. 1B).

Nine hundred and nineteen individuals out of 4557 individuals (20.2%) diagnosed with Alzheimer's disease dementia were false positives, meaning they were ADNC-negative. In a subset of 4474 patients with available data on onset of symptoms, we assessed whether clinical-pathological discordance differed by age at onset. The proportion of Alzheimer's disease dementia cases lacking ADNC differed significantly between early- and late-onset groups (*P* < 0.0001; Fig. 1D). Specifically, false-positive Alzheimer's disease dementia was observed in 6.9% of early-onset cases (75/1089) compared to 23.8% of late-onset cases (805/3385), indicating a substantially higher frequency of ADNC-negative cases among late-onset individuals.

### Frequency of co-pathologies across clinical groups

We calculated the frequency of the neuropathological findings identified at autopsy in individuals diagnosed with clinical Alzheimer's disease dementia, PCA and lvPPA (Fig. 1C). Each cell in the heatmap shows the percentage of participants within a given clinical diagnosis group who exhibited the specified co-pathology or met the indicated neuropathologic threshold. We found that 79.8% of individuals with amnestic Alzheimer's disease dementia showed ADNC as defined by moderate to frequent neuritic plaques and tangles in Braak stages III–VI; the frequency for individuals with atypical Alzheimer's disease diagnoses was 91.5–96.2%. Two-sample proportion tests showed that the frequency of ADNC was statistically different between amnestic and atypical Alzheimer's disease diagnoses, although only the difference between amnestic Alzheimer's disease dementia and PCA survived correction for multiple comparisons (*P* = 0.04). Between 20.8 and 50.9% of individuals with both amnestic and atypical Alzheimer's disease displayed CAA pathology and between 35.6 and 40.0% displayed LB pathology. Furthermore, 35% of individuals diagnosed with Alzheimer's disease dementia met the neuropathologic criteria for LATE-NC and 4.8% for PART. Frequency of PART was higher in amnestic Alzheimer's disease compared to both lvPPA and PCA (*P* = 0.03). *P*-values for comparisons in frequency between amnestic and atypical Alzheimer's disease diagnoses are listed in Supplementary Table 5. Analyses were replicated in early- and late-onset subgroups (Supplementary Fig. 2 and Table 6).

### Positive predictive value and frequency of co-pathologies in early-onset and late-onset dementia

**Figure 2:**
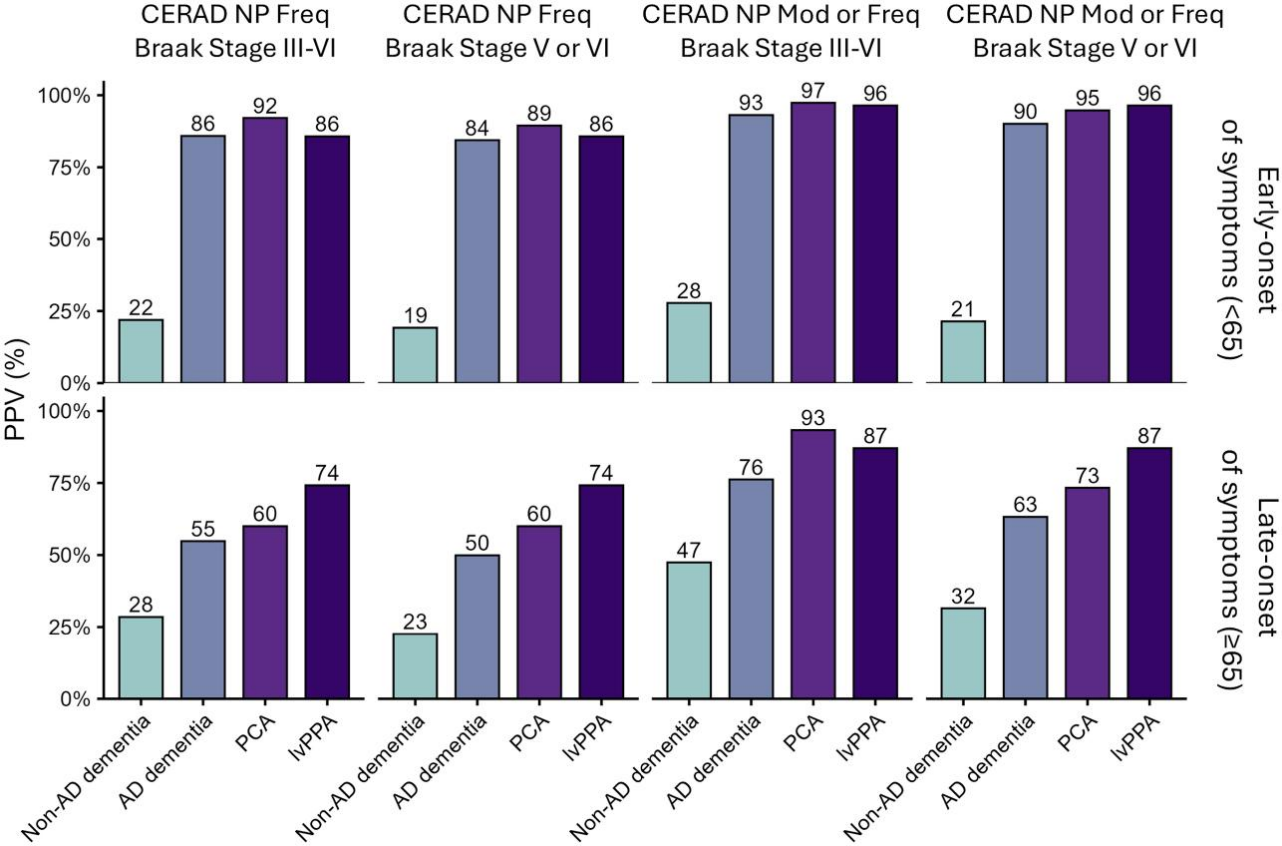
**PPV of clinical diagnoses in individuals with early- versus and late-onset of symptoms.** PPV for each clinical diagnosis group (amnestic AD, PCA, lvPPA and non-AD dementia; *N* = 6259), stratified by neuropathological threshold, in individuals with early-onset (<65 years old, *N* = 1968) versus late-onset (≥65 years old, *N* = 4291). Thresholds are defined by combinations of CERAD neuritic plaque frequency (moderate or frequent) and Braak stage (III–VI or V–VI). AD, Alzheimer’s disease; CERAD NP, Consortium to Establish a Registry for Alzheimer’s Disease neuritic plaque; Freq, frequent; lvPPA, logopenic variant primary progressive aphasia; Mod, moderate; PCA, posterior cortical atrophy; PPV, positive predictive value.

We calculated the PPV across clinical diagnoses, stratified by histopathological severity and onset of symptoms (early- versus late-onset; Fig. 2). PPV estimates and 95% CIs are provided in Supplementary Table 7. Overall, the PPV for the clinical diagnosis of probable Alzheimer's disease dementia was higher in early-onset compared to late-onset cases, ranging from 84 to 93% in early-onset patients across thresholds, with the highest values observed for the moderate or frequent neuritic plaques with Braak stages III–VI threshold. For PCA and lvPPA, PPV varied according to both onset group and neuropathological threshold. In general, PPV values were lower in late-onset cases for these atypical presentations, particularly when using more stringent thresholds (i.e. frequent neuritic plaques with Braak stages V–VI). However, for the moderate or frequent neuritic plaques with Braak stages III–VI threshold, PPV values for lvPPA and PCA were similar between early- and late-onset groups.

### Risk of false-positive Alzheimer’s disease dementia diagnosis

**Figure 3:**
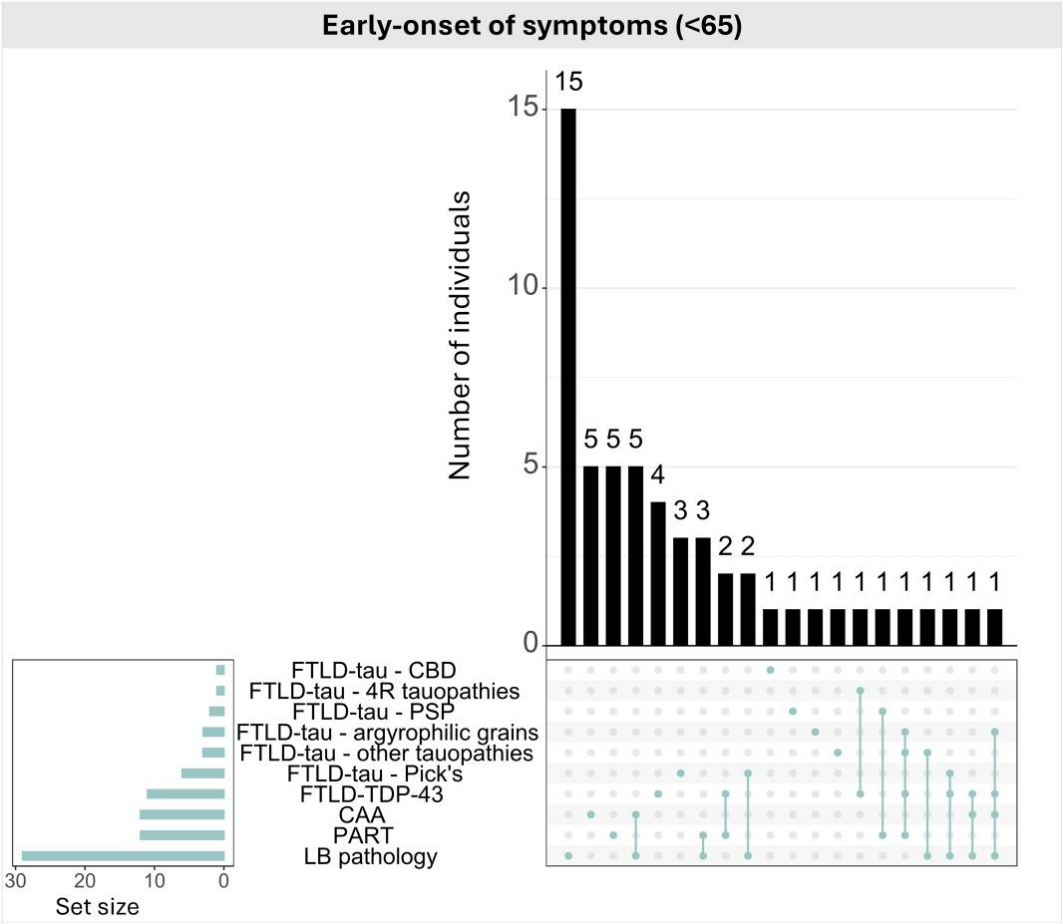
**Co-pathology burden among clinically diagnosed Alzheimer's disease individuals with early-onset of symptoms who were negative for ADNC (i.e. false positives, *N* = 75).** UpSet plots showing the number of individuals exhibiting different combinations of co-pathologies in the early-onset group. The vertical bars indicate the number of individuals exhibiting each specific combination of co-pathologies, while the connected dots below the bars indicate which co-pathologies are present in that combination. Single, isolated dots represent individuals with only one pathological finding, whereas connected dots represent co-occurring pathologies. The horizontal bars on the left show the total number of individuals with each pathology, regardless of combinations, providing a reference for frequency. CAA, cerebral amyloid angiopathy; CBD, corticobasal degeneration; FTLD-tau, frontotemporal lobar degeneration with tau pathology; FTLD-TDP-43, frontotemporal lobar degeneration with TDP-43 pathology; LB, Lewy body pathology; PART, primary age-related tauopathy; PSP, progressive supranuclear palsy; 4R, four-repeat tau.

**Figure 4:**
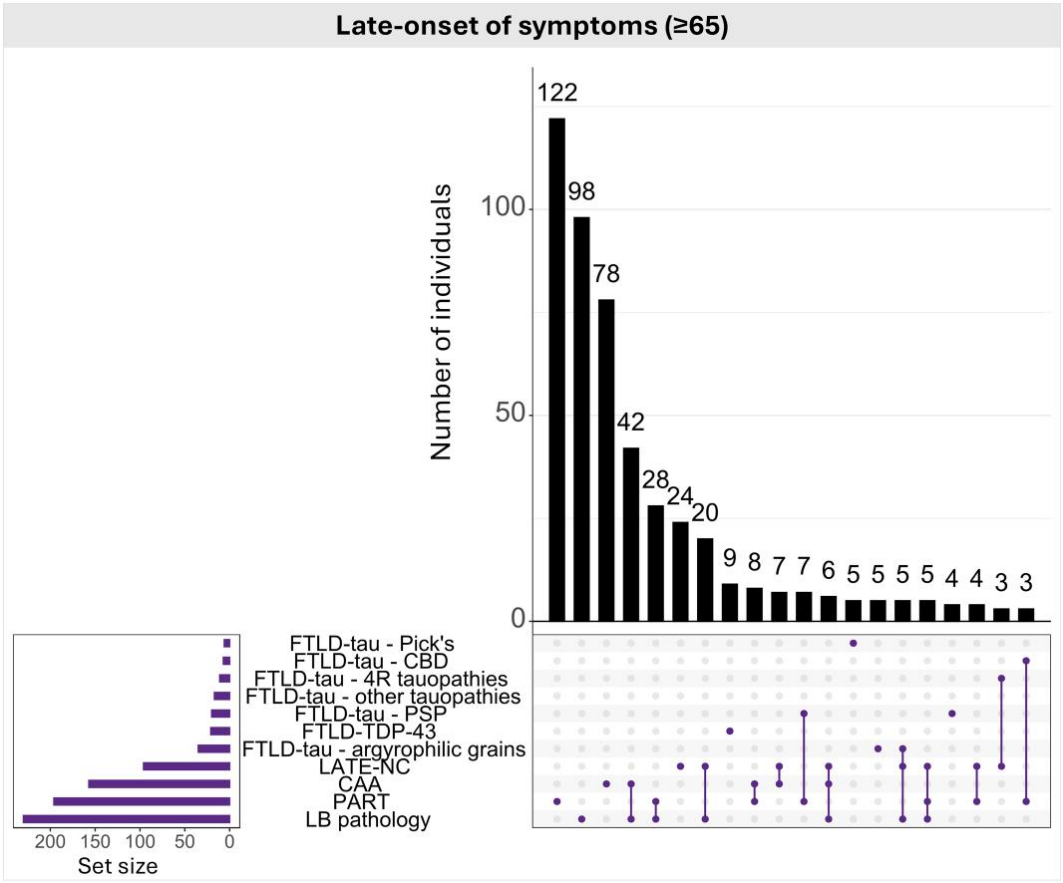
**Co-pathology burden among clinically diagnosed Alzheimer's disease individuals with late-onset of symptoms who were negative for ADNC (i.e. false positives, *N* = 805).** UpSet plots showing the number of individuals exhibiting different combinations of co-pathologies in the late-onset group. The vertical bars indicate the number of individuals exhibiting each specific combination of co-pathologies, while the connected dots below the bars indicate which co-pathologies are present in that combination. Single, isolated dots represent individuals with only one pathological finding, whereas connected dots represent co-occurring pathologies. The horizontal bars on the left show the total number of individuals with each pathology, regardless of combinations, providing a reference for frequency. CAA, cerebral amyloid angiopathy; CBD, corticobasal degeneration; FTLD-tau, frontotemporal lobar degeneration with tau pathology; FTLD-TDP-43, frontotemporal lobar degeneration with TDP-43 pathology; LB, Lewy body pathology; LATE-NC, limbic-predominant age-related TDP-43 encephalopathy neuropathologic change; PART, primary age-related tauopathy; PSP, progressive supranuclear palsy; 4R, four-repeat tau.

Figures 3 and 4 illustrate the combinations and raw counts of co-pathologies observed among false-positive Alzheimer's disease dementia cases in the early-onset group and late-onset group, while the corresponding values and percentages are listed in Supplementary Table 8.

In the early-onset group, we found that LB pathology was the most common pathology observed in false-positive Alzheimer's disease dementia cases (20.0%), followed by FTLD-TDP-43 (11.4%) and then CAA (6.9%) (Fig. 3). In the late-onset group, we found that PART was the most common pathology observed in false-positive Alzheimer's disease dementia cases (15.2%), followed by LB pathology (12.2%), CAA (9.8%), LATE-NC (7.4%) and a combination of CAA and LB pathology (5.3%) (Fig. 4).

**Figure 5:**
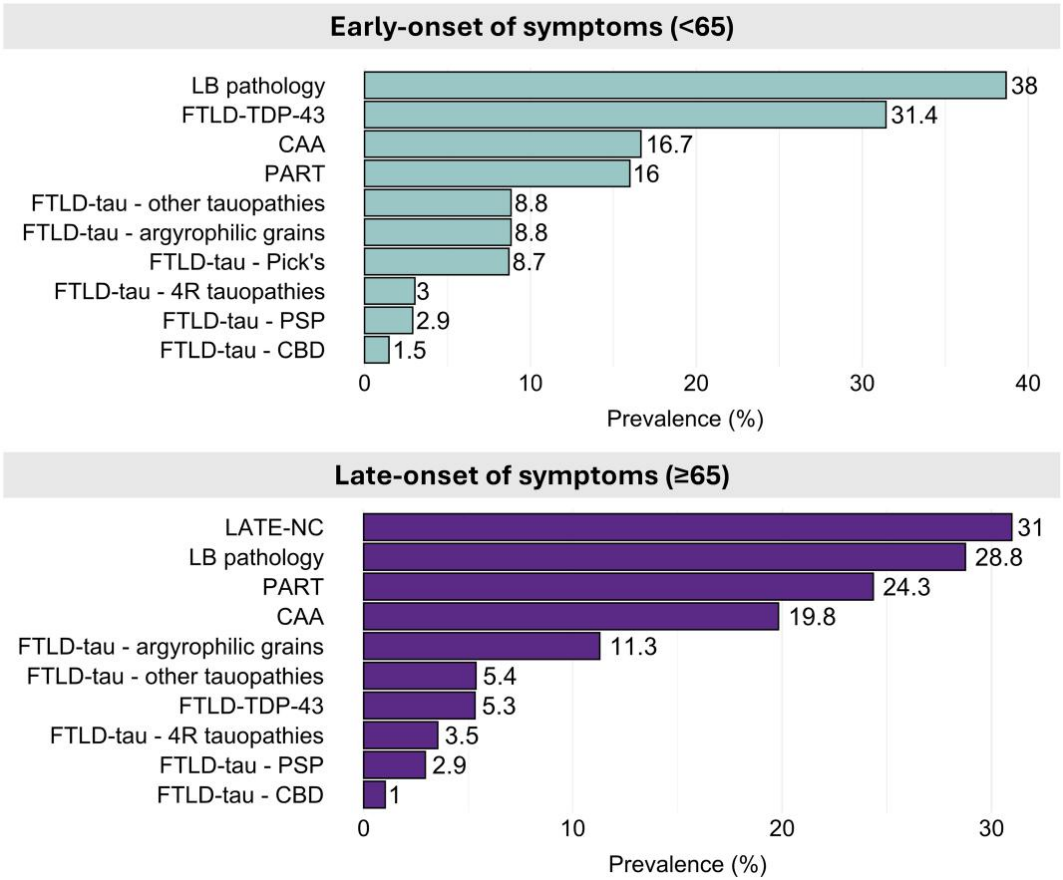
**Marginal frequency of different co-pathologies among clinically diagnosed Alzheimer's disease individuals who were negative for ADNC (i.e. false positives, *N* = 880).** The top panel shows the marginal frequency of the different co-pathologies in the early-onset group (*N* = 75), and the bottom panel shows the marginal frequency of the different co-pathologies in the late-onset group (*N* = 805). CAA, cerebral amyloid angiopathy; CBD, corticobasal degeneration; 4R, four-repeat tau; FTLD-TDP-43, frontotemporal lobar degeneration with TDP-43 pathology; FTLD-tau, frontotemporal lobar degeneration with tau pathology; LB, Lewy body pathology; LATE-NC, limbic-predominant age-related TDP-43 encephalopathy neuropathologic change; PART, primary age-related tauopathy; PSP, progressive supranuclear palsy.

We then computed the marginal frequency of individual pathologies to capture their overall frequency independent of co-occurring combinations. We found that vascular pathology was observed in 93% of early-onset false-positive Alzheimer's disease dementia cases, followed by LB pathology (38.7%) and FTLD-TDP-43 (31.4%) (Fig. 5, top panel; Supplementary Table 9). In the late-onset group, marginal frequency calculations showed that vascular pathology was present 98% of the false-positive cases, followed by LATE-NC (31%) and LB pathology (28.8%) (Fig. 5, bottom panel; Supplementary Table 6).

**Figure 6:**
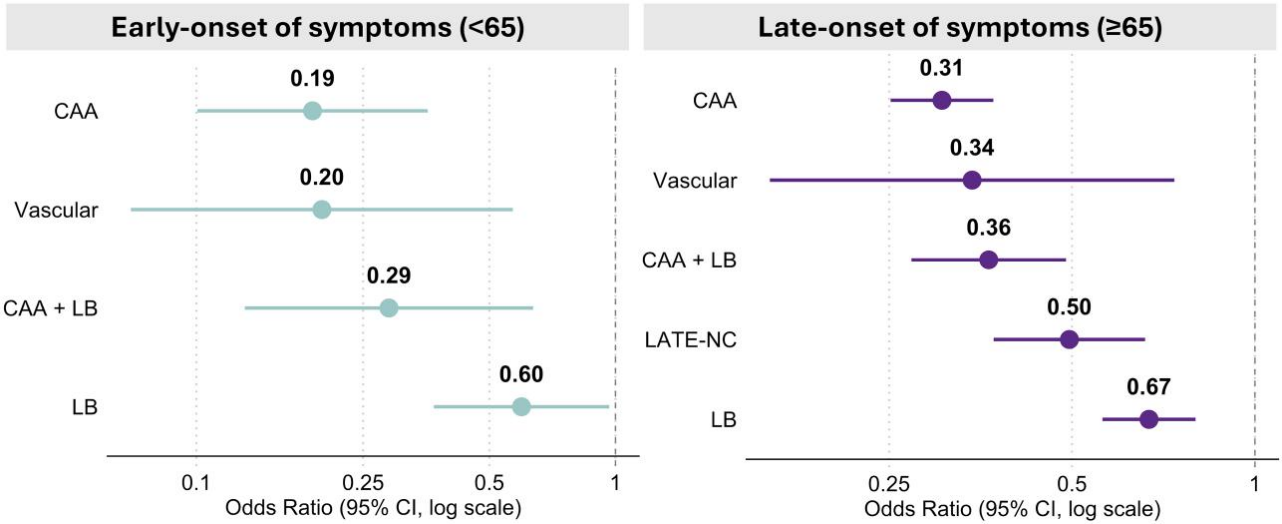
**Co-pathology burden among clinically diagnosed Alzheimer's disease individuals who were negative for ADNC (i.e. false positives, *N* = 880).** Forest plot of ORs with 95% CIs for the association between co-pathologies and the likelihood of being classified as a false-positive Alzheimer's disease dementia case compared to a true-positive Alzheimer's disease dementia case in individuals with early-onset of symptoms (left; *N* = 75) and late-onset of symptoms (right; *N* = 805). ORs are shown on a logarithmic scale. OR > 1 indicates increased odds of false-positive diagnosis among individuals with the co-pathology compared to a true-positive diagnosis, while OR < 1 indicates reduced odds. The dashed vertical line indicates the null value (OR = 1, no association). CAA, cerebral amyloid angiopathy; LATE-NC, limbic-predominant age-related TDP-43 encephalopathy neuropathologic changes; LB, Lewy body pathology.

We assessed how the presence of the most prevalent co-pathologies, as well as vascular pathology burden, was associated with false-positive clinical diagnoses of Alzheimer's disease dementia. Due to the small number of FTLD-TDP-43 assessment (*N* = 35), this group was excluded from the primary OR analysis to avoid unstable estimates. In individuals with early-onset of symptoms, we found significant associations with true-positive Alzheimer's disease dementia diagnoses compared to false-positive Alzheimer's disease dementia diagnoses, suggesting that these pathologies are more likely to be present in the presence of ADNC rather than the absence, in individuals with Alzheimer's disease dementia (CAA: OR = 0.189, 95% CI: 0.101–0.357; vascular pathology: OR = 0.199, 95% CI: 0.0698–0.569; CAA + LB: OR = 0.288, 95% CI: 0.131–0.636; LB: OR = 0.597, 95% CI: 0.369–0.966; Fig. 6, left panel). In individuals with late-onset of symptoms, we found once again significant associations with true-positive Alzheimer's disease dementia diagnoses compared to false-positive Alzheimer's disease dementia diagnoses (CAA: OR = 0.305, 95% CI: 0.251–0.371; vascular pathology: OR = 0.342, 95% CI: 0.159–0.737; CAA + LB: OR = 0.364, 95% CI: 0.272–0.489; LB: OR = 0.0.670, 95% CI: 0.561–0.799; LATE-NC: OR = 0.495, 95% CI: 0.372–0.660; Fig. 6, right panel).

### Positive predictive value and negative predictive value of clinical diagnoses for biomarker severity *in vivo*

**Figure 7:**
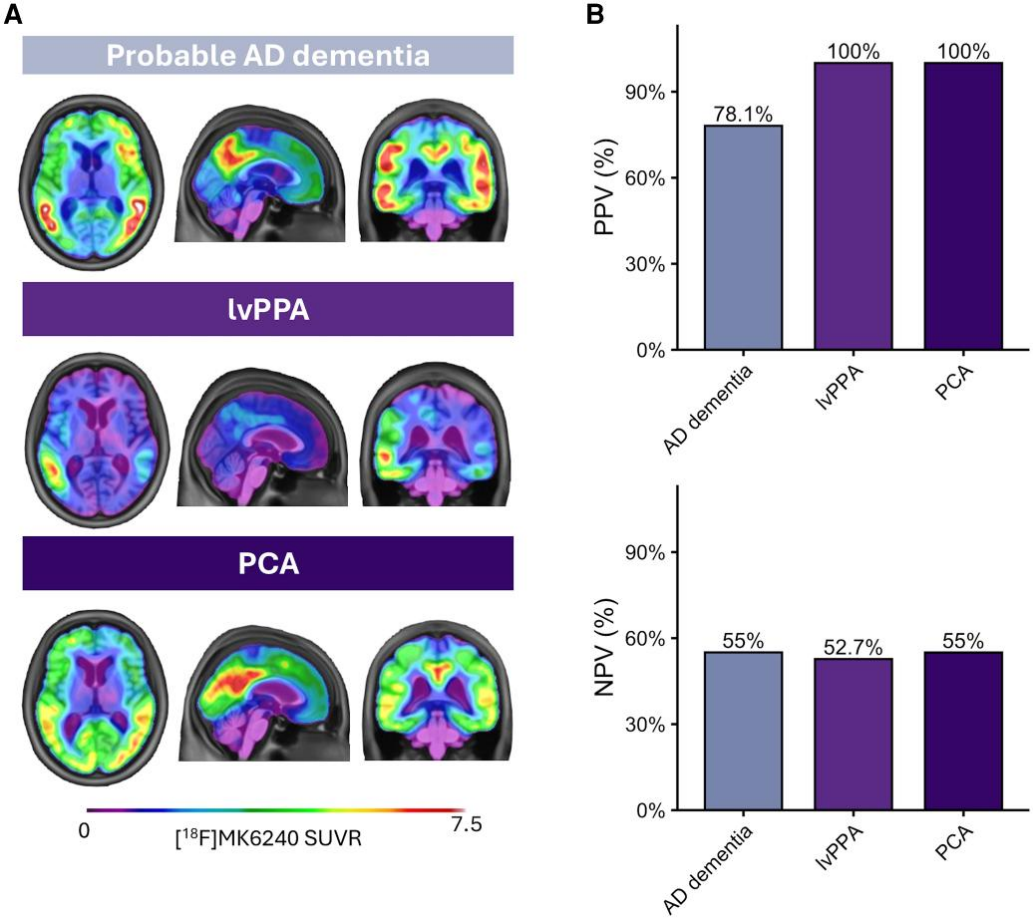
***In vivo* replication of clinical-biological concordance in the TRIAD cohort (*N* = 76).** (**A**) Representative tau-PET images of patients diagnosed with probable Alzheimer's disease dementia (top), PCA (middle) and lvPPA (bottom). Colour bar represents tau-PET SUVR. (**B**) PPV and NPV of the clinical diagnosis across diagnostic groups (AD dementia, *N* = 32; lvPPA, *N* = 16; PCA, *N* = 28), reflecting the extent to which clinical syndromes predict the presence (A+T+) or absence (A−T−) of underlying Alzheimer’s disease pathology *in vivo*. lvPPA, logopenic variant primary progressive aphasia; PCA, posterior cortical atrophy; AD, Alzheimer’s disease.

We first illustrate representative brain scans from TRIAD participants with amnestic Alzheimer's disease dementia, lvPPA and PCA, highlighting the typical tau-PET patterns observed in each diagnosis (Fig. 7A). We assessed the frequency of biomarker profiles within each clinical diagnosis group of the TRIAD cohort (Fig. 7B). We calculated the PPV and NPV across diagnoses (clinical Alzheimer's disease dementia, lvPPA or PCA) for the A+T+ status (Fig. 7C). PPV and NPV estimates and 95% CIs are provided in Supplementary Table 10. More precisely, the PPV represents the probability that an individual with a given clinical diagnosis is A+T+ on PET, while the NPV represents the probability that an individual without a given clinical diagnosis is A−T− on PET. The PPV for the clinical diagnosis of probable Alzheimer's disease dementia was 78.1%, and the PPV for lvPPA and PCA was 100%. The NPV for the clinical diagnosis of probable Alzheimer's disease dementia was 55.0%, and the NPV for lvPPA and PCA was 52.7 and 55%, respectively. We found a difference in PPV between probable Alzheimer's disease dementia and PCA (*P* = 0.0353), but not between probable Alzheimer's disease dementia and lvPPA (*P* = 0.118). We did not find significant differences in NPV between probable Alzheimer's disease dementia and PCA (*P* = 0.819) or between probable Alzheimer's disease dementia and lvPPA (*P* = 0.819).

## Discussion

This study investigated the accuracy of clinical diagnoses in predicting underlying Alzheimer's disease neuropathology across amnestic and atypical Alzheimer's disease phenotypes, as well as the frequency of co-pathologies and its impact on diagnostic specificity. Using data from both an autopsy database and an observational *in vivo* cohort study, we found that lvPPA and PCA clinical syndromes showed a higher PPV for underlying Alzheimer's disease pathology than typical amnestic Alzheimer's disease diagnoses, especially in late-onset AD. We also observed that vascular pathology and LATE-NC were the most common pathologies in individuals with a clinical diagnosis of probable Alzheimer's disease but lacking significant ADNC. Together, our results suggest that the atypical Alzheimer's disease phenotypes lvPPA and PCA, while less common, may provide greater diagnostic confidence, whereas false-positive Alzheimer's disease dementia diagnoses are more frequent and often attributable to a variety of underlying pathological processes.

First, we calculated PPVs to assess how the different clinical diagnoses reflect biological Alzheimer's disease as measured by neuropathological assessments. We found that across different neuropathological thresholds, the diagnosis of lvPPA and PCA had higher PPVs for Alzheimer's disease neuropathology than probable AD. Our results suggest that, when clinically assigned, lvPPA and PCA phenotypes carry a higher likelihood of underlying Alzheimer's disease neuropathology than the more common amnestic presentation of AD. With increasing availability and use of reliable biomarkers such as p-tau217,^28^ clinicians are often faced with interpreting results in individual patients, where information such as age and clinical syndrome are critical to confidently rule in AD.^29^ Our results show that the identification of lvPPA or PCA significantly increases the likelihood of underlying Alzheimer's disease pathology, providing valuable context for interpreting biomarker results without the need for confirmatory PET or CSF testing.

We also found that the PPV for amnestic Alzheimer's disease differed by age at symptom onset. The PPV of probable Alzheimer's disease for various levels of ADNC was higher in individuals with early-onset dementia. In contrast, the PPV of probable Alzheimer's disease was lower in late-onset dementia, reflecting a greater proportion of clinical Alzheimer's disease diagnoses without ADNC, i.e. a higher frequency of false-positive cases. This finding reflects the fewer neurodegenerative aetiologies that can cause the early-onset amnestic dementia phenotype.^5^ In contrast, the larger number of neurodegenerative diseases associated with late-onset amnestic dementia reflects the lower specificity of the probable Alzheimer's disease syndrome for advanced ADNC.

We next investigated neuropathological findings in individuals who received a clinical Alzheimer's disease diagnosis but did not meet pathological criteria for ADNC. LATE-NC emerged as a highly relevant co-pathology in false-positive Alzheimer's disease dementia cases in the late-onset group. More precisely, LATE-NC occurred in 31% of individuals with the probable Alzheimer's disease diagnosis but without advanced ADNC. These results suggest that LATE-NC are commonly observed in older individuals with a clinical diagnosis of Alzheimer's disease dementia who lack advanced ADNC, consistent with growing evidence identifying LATE as a major age-related proteinopathy that can closely mimic amnestic AD. Clinical criteria for possible and probable LATE have recently been proposed,^30^ highlighting that not all amnestic cognitive impairment is attributable to ADNC. Given that these criteria were published in 2025, awareness and formal operationalization of LATE as a distinct clinicopathological entity were limited during the present study period, with all clinical assessments occurring prior to the publication of LATE clinical criteria. This temporal context is relevant when interpreting clinicopathological associations. In this context, future studies that incorporate formal differentiation between probable/possible LATE and probable/possible Alzheimer's disease in clinical diagnostic frameworks will be essential to refine PPVs of clinical Alzheimer's disease syndromes for ADNC at autopsy, particularly in older populations where mixed and age-related pathologies are highly prevalent.

PART also emerged as a frequent pathology among individuals with a false-positive diagnosis of Alzheimer's disease dementia in the late-onset group. This observation is consistent with the topographical distribution of PART pathology, which predominantly involves the medial temporal lobe and can give rise to an amnestic clinical presentation that can resemble probable AD,^3,31,32^ thereby increasing the likelihood of a misdiagnosis. These findings also highlight the importance of Aβ biomarkers, as their assessment can help distinguish Alzheimer's disease from PART and other tauopathies, reducing the risk of misdiagnosis. However, the interpretation of PART in the context of clinical-pathological discordance warrants careful consideration, due to substantial definitional overlap with Alzheimer's disease neuropathologic criteria. Specifically, PART is defined by low levels of Aβ pathology and limited tau deposition, which partially intersects with the thresholds used to define ADNC in our study. As a result, cases classified as PART may inherently cluster within ADNC-negative groups, limiting the ability to determine whether PART independently contributes to clinical-pathological mismatch or instead reflects a boundary category within existing staging frameworks. Furthermore, it is important to note that the clinical significance of PART remains a matter of ongoing debate.^33^ PART is often considered to reflect age-related tau accumulation rather than a distinct clinical syndrome, as it has been shown that ∼55% of individuals with PART are CU.^34^ As such, the clinical significance of PART in individuals lacking ADNC should be interpreted with caution. Taken together, these findings suggest that while PART is highly prevalent in late-onset individuals with clinical Alzheimer's disease without ADNC, its role in clinical-pathological discordance is constrained by definitional overlap with ADNC criteria and may reflect a continuum of age-related medial temporal lobe tau pathology rather than a distinct source of diagnostic mismatch.

Interestingly, among individuals with a clinical diagnosis of probable AD, the pathologies that were associated with false-positive cases (i.e. LATE-NC, LB and CAA) were more common in cases with high ADNC than in those without. These results converge with several studies showing that these pathologies may arise more frequently in the context of concomitant ADNC,^35-37^ where they may be more involved in exacerbating cognitive decline rather than producing a mimic of amnestic Alzheimer's disease on their own.

Finally, we evaluated whether lvPPA and PCA clinical phenotypes retain differential predictive value for biologically defined Alzheimer's disease when assessed *in vivo* using PET biomarkers. Using data from the TRIAD cohort, we were able to replicate autopsy findings and found that the PPV of atypical syndromes was higher than that of amnestic Alzheimer's disease dementia. With increasing availability of fluid biomarkers such as plasma p-tau217 and established CSF/PET measures of Aβ and tau, Alzheimer's disease diagnosis is increasingly performed in a biomarker-informed context. In this setting, syndrome-level information remains critical, as our findings suggest that the identification of lvPPA or PCA further increases the pre-test probability of underlying Alzheimer's disease pathology and may meaningfully influence the interpretation of biomarker results, particularly when confirmatory PET or CSF testing is not available. However, it is important to emphasize that the ‘probable AD’ diagnosis examined in our study is, by definition, determined in the absence of Alzheimer's disease biomarkers. Therefore, our findings of higher PPV for PCA and lvPPA are most relevant for centres in which confirmatory Alzheimer's disease biomarker testing is not available. Nonetheless, *in vivo* assessment of misdiagnosis remains challenging, as biomarkers for most non-AD pathologies are not yet available. PART represents a potential exception, since tau-PET tracers should theoretically bind to neurofibrillary tangles characteristic of both Alzheimer's disease and PART; however, it remains unclear whether tau-PET can reliably detect it, particularly given the lower tangle burden typically observed in PART.^38-41^ The development of biomarkers for non-AD pathologies will be essential for accurately characterizing co-pathologies *in vivo* and understanding their contribution to clinical presentations, particularly in older individuals presenting with amnestic syndromes. Our findings provide a reference point against which the added value of emerging biomarkers for non-AD proteinopathies can be assessed in the future.

This study should be interpreted in the context of its limitations. First, both the *in vivo* and post-mortem cohorts were composed of individuals who volunteered for research on neurodegenerative disease and thus may not fully capture the demographic and clinical diversity of the general population at risk for dementia in North America. Second, because missing values in non-target pathologies were retained in the denominator when calculating the frequency, the resulting percentages may slightly overestimate the true frequency of co-pathologies. Some participants with missing data could theoretically have additional co-pathologies, so the observed proportions should be interpreted as approximate estimates rather than exact values. Third, we were limited to the presence or absence of pathologies at autopsy and could not determine which represented the primary neuropathological finding, which may have further influenced clinicopathological interpretations. Fourth, although the replication in the TRIAD cohort leveraged *in vivo* amyloid- and tau-PET biomarkers as surrogates of Alzheimer's disease pathology, this approach is inherently not equivalent to post-mortem neuropathological confirmation. However, tau-PET analyses were based on a meta-ROI intended to capture biologically meaningful intermediate-to-advanced tau pathology. In these later stages, tau-PET signal closely approximates true pathological burden, supporting its use as a surrogate for biologically defined Alzheimer's disease *in vivo*. Fifth, the NACC and TRIAD cohorts differed substantially in the representation of atypical Alzheimer's disease phenotypes, with lvPPA and PCA comprising only 1–1.3% and 1.1% of NACC Alzheimer's disease cases, respectively, versus 21% and 36.8% in TRIAD. These differences likely reflect the distinct nature of the cohorts: NACC is a large, multi-centre database in which atypical presentations may be underdiagnosed, whereas TRIAD is a specialized memory clinic enriched for atypical Alzheimer's disease phenotypes. Additional differences in age at death and sex distribution further distinguish the cohorts, which should be considered when comparing results across populations. Sixth, in the NACC cohort, the average interval between the last clinical assessment and death was approximately 22 months, raising the possibility that some individuals with atypical Alzheimer's disease phenotypes may have experienced clinical evolution towards a more amnestic presentation closer to death that was not captured by the final visit. This may have led, in some cases, to classification based on a later-stage global dementia phenotype rather than the initial syndromic presentation and could therefore influence estimates of PPV for lvPPA and PCA diagnoses. However, the replication of these findings in the TRIAD cohort, in which *in vivo* biomarker assessments and clinical diagnosis were make within a shorter time frame, helps attenuate this concern. Seventh, while our results highlight the higher specificity of PCA and lvPPA clinical syndromes for ADNC, these rarer clinical variants are often diagnosed later in the disease course, in specialized settings such as memory clinics. Therefore, the clinical utility of these findings may be limited to specialized centres. Finally, we did not include other atypical forms of AD, including the frontal variant and cases presenting with CBS, which may exhibit distinct pathological or clinical profiles. Future studies should aim to include these less common presentations to determine whether the observed associations extend to the broader spectrum of Alzheimer's disease phenotypes.

In summary, while co-pathologies are common in individuals with amnestic Alzheimer's disease and can complicate the link between symptoms and underlying ADNC, our findings suggest that this is not the case for atypical variants such as PCA and lvPPA. In these phenotypes, Alzheimer's disease pathology was consistently present, indicating that clinical diagnosis in such cases carries a high degree of diagnostic confidence, even in the absence of confirmatory biomarkers.

## Supplementary Material

fcag304_Supplementary_Data

## Data Availability

Data from the National Alzheimer’s Coordinating Center (NACC) used in this study can be accessed upon request at https://naccdata.org/, following approval of a data use agreement. Requests for TRIAD cohort data and related materials will be evaluated by McGill University to ensure compliance with any existing intellectual property or confidentiality requirements. Reasonable requests for access to anonymized data for the purpose of replication may be directed to the corresponding authors and will be considered in accordance with institutional policies. The analyses presented in this study did not require the development of specialized code beyond standard statistical procedures.
