# Supplementary material for: Posterior cortical atrophy and logopenic variant primary progressive aphasia are more specific for Alzheimer’s disease pathology than probable Alzheimer’s disease
Source: Brain Commun. 2026 Aug 6;8(4):fcag304. doi: 10.1093/braincomms/fcag304 (PMC13457939; doi:10.1093/braincomms/fcag304)
Supplement: Supplementary file 1 [file fcag304_supplementary_data.pdf]

Supplementary Materials

Trudel, Therriault, Macedo et al.

Clinical diagnosis	CERAD NP Freq & Braak III–VI	CERAD NP Freq & Braak V–VI	CERAD NP Mod or Freq & Braak III–VI	CERAD NP Mod or Freq & Braak V–VI
**AD dementia**	61.6% (60.2–63)	57.6% (56.1–59)	79.8% (78.6–81)	69.1% (67.7–70.4)
**PCA**	83% (70.8–90.8)	81.1% (68.6–89.4)	96.2% (87.2–99)	88.7% (77.4–94.7)
**lvPPA**	79.7% (67.7–88)	79.7% (67.7–88)	91.5% (81.6–96.3)	91.5% (81.6–96.3)
**Non-AD dementia**	25.2% (23.2–27.3)	20.8% (19–22.8)	38.1% (35.8–40.4)	26.5% (24.5–28.7)

Supplementary Table 1. Positive predictive value (PPV) estimates and 95% confidence intervals across diagnoses and stratified by histopathological severity.

ADNC threshold	Group 1	Group 2	χ^2	p-value	Adj. p-value (FDR)
**CERAD NP Freq & Braak V-VI**	AD dementia	PCA	10.99	0.00092	0.00158
**CERAD NP Freq & Braak V-VI**	AD dementia	lvPPA	10.78	0.00103	0.00165
**CERAD NP Freq & Braak V-VI**	AD dementia	Non-AD dementia	414.37	<0.001	<0.001
**CERAD NP Freq & Braak V-VI**	PCA	lvPPA	0	1	1
**CERAD NP Freq & Braak V-VI**	PCA	Non-AD dementia	110.70	<0.001	<0.001
**CERAD NP Freq & Braak V-VI**	lvPPA	Non-AD dementia	115.80	<0.001	<0.001
**CERAD NP Mod or Freq & Braak V-VI**	AD dementia	PCA	8.54	0.00347	0.0049
**CERAD NP Mod or Freq & Braak V-VI**	AD dementia	lvPPA	12.75	0.00036	0.000665
**CERAD NP Mod or Freq & Braak V-VI**	AD dementia	Non-AD dementia	495.32	<0.001	<0.001
**CERAD NP Mod or Freq & Braak V-VI**	PCA	lvPPA	0.035	0.85137	0.888
**CERAD NP Mod or Freq & Braak V-VI**	PCA	Non-AD dementia	83.64	<0.001	<0.001
**CERAD NP Mod or Freq & Braak V-VI**	lvPPA	Non-AD dementia	100.66	<0.001	<0.001
**CERAD NP Freq & Braak III-VI**	AD dementia	PCA	9.32	0.00227	0.0034
**CERAD NP Freq & Braak III-VI**	AD dementia	lvPPA	7.32	0.00681	0.0086
**CERAD NP Freq & Braak III-VI**	AD dementia	Non-AD dementia	358.56	<0.001	<0.001
**CERAD NP Freq & Braak III-VI**	PCA	lvPPA	0.045	0.832	0.888
**CERAD NP Freq & Braak III-VI**	PCA	Non-AD dementia	78.20	<0.001	<0.001
**CERAD NP Freq & Braak III-VI**	lvPPA	Non-AD dementia	76.56	<0.001	<0.001
**CERAD NP Mod or Freq & Braak III-VI**	AD dementia	PCA	7.81	0.00519	0.00692
**CERAD NP Mod or Freq & Braak III-VI**	AD dementia	lvPPA	4.27	0.03879	0.0465
**CERAD NP Mod or Freq & Braak III-VI**	AD dementia	Non-AD dementia	391.06	<0.001	<0.001
**CERAD NP Mod or Freq & Braak III-VI**	PCA	lvPPA	0.40	0.52527	0.6
**CERAD NP Mod or Freq & Braak III-VI**	PCA	Non-AD dementia	46.91	<0.001	<0.001
**CERAD NP Mod or Freq & Braak III-VI**	lvPPA	Non-AD dementia	42.49	<0.001	<0.001

Supplementary Table 2. Chi-squared tests comparing PPVs across clinical groups. This table shows the results of Chi-squared tests assessing whether the proportion of participants with confirmed AD neuropathologic change (i.e., the PPV) differs between clinical diagnosis groups (Non-AD dementia, AD dementia, PCA, lvPPA,). P-values were adjusted for multiple comparisons using the false discovery rate (FDR). Significant results indicate that the likelihood of a clinical diagnosis accurately predicting AD neuropathology varies across groups.

Clinical diagnosis	CERAD NP Freq & Braak III–VI	CERAD NP Freq & Braak V–VI	CERAD NP Mod or Freq & Braak III–VI	CERAD NP Mod or Freq & Braak V–VI
AD dementia	78.2% (76.6–79.7)	83.1% (81.7–84.5)	65.9% (64.1–67.6)	78.6% (77–80)
PCA	54% (52.9–55.2)	58.4% (57.3–59.5)	38% (36.9–39.2)	49.5% (48.4–50.7)
lvPPA	54% (52.9–55.2)	58.4% (57.3–59.5)	38% (36.9–39.1)	49.6% (48.5–50.7)
Non-AD dementia	47.5% (46.2–48.8)	51.9% (50.6–53.2)	30.6% (29.5–31.9)	42.1% (40.8–43.4)

Supplementary Table 3. Negative predictive value (NPV) estimates and 95% confidence intervals across diagnoses and stratified by histopathological severity.

ADNC threshold	Group 1	Group 2	χ^2	p-value	Adj. p-value (FDR)
**CERAD NP Freq & Braak V-VI**	AD dementia	PCA	10.99	0.00092	0.00157714
**CERAD NP Freq & Braak V-VI**	AD dementia	lvPPA	10.78	0.00103	0.001648
**CERAD NP Freq & Braak V-VI**	AD dementia	Non-AD dementia	666.164	<0.001	<0.001
**CERAD NP Freq & Braak V-VI**	PCA	lvPPA	0	1	1
**CERAD NP Freq & Braak V-VI**	PCA	Non-AD dementia	103.169	<0.001	<0.001
**CERAD NP Freq & Braak V-VI**	lvPPA	Non-AD dementia	108.701	<0.001	<0.001
**CERAD NP Mod or Freq & Braak V-VI**	AD dementia	PCA	8.544	0.00347	0.00489882
**CERAD NP Mod or Freq & Braak V-VI**	AD dementia	lvPPA	12.752	<0.001	<0.001
**CERAD NP Mod or Freq & Braak V-VI**	AD dementia	Non-AD dementia	915.543	<0.001	<0.001
**CERAD NP Mod or Freq & Braak V-VI**	PCA	lvPPA	0.035	0.85137	0.88838609
**CERAD NP Mod or Freq & Braak V-VI**	PCA	Non-AD dementia	94.68	<0.001	<0.001
**CERAD NP Mod or Freq & Braak V-VI**	lvPPA	Non-AD dementia	114.649	<0.001	<0.001
**CERAD NP Freq & Braak III-VI**	AD dementia	PCA	9.318	0.00227	0.003405
**CERAD NP Freq & Braak III-VI**	AD dementia	lvPPA	7.323	0.00681	0.00860211
**CERAD NP Freq & Braak III-VI**	AD dementia	Non-AD dementia	652.697	<0.001	<0.001
**CERAD NP Freq & Braak III-VI**	PCA	lvPPA	0.045	0.832	0.88838609
**CERAD NP Freq & Braak III-VI**	PCA	Non-AD dementia	84.316	<0.001	<0.001
**CERAD NP Freq & Braak III-VI**	lvPPA	Non-AD dementia	82.959	<0.001	<0.001
**CERAD NP Mod or Freq & Braak III-VI**	AD dementia	PCA	7.811	0.00519	0.00692
**CERAD NP Mod or Freq & Braak III-VI**	AD dementia	lvPPA	4.27	0.03879	0.046548
**CERAD NP Mod or Freq & Braak III-VI**	AD dementia	Non-AD dementia	996.319	<0.001	<0.001
**CERAD NP Mod or Freq & Braak III-VI**	PCA	lvPPA	0.404	0.52527	0.60030857
**CERAD NP Mod or Freq & Braak III-VI**	PCA	Non-AD dementia	70.101	<0.001	<0.001
**CERAD NP Mod or Freq & Braak III-VI**	lvPPA	Non-AD dementia	65.728	<0.001	<0.001

Supplementary Table 4. Chi-squared tests comparing NPVs across clinical groups. This table shows the results of Chi-squared tests assessing whether the proportion of participants without confirmed AD neuropathologic change (i.e., the NPV) differs between clinical diagnosis groups (Non-AD dementia, AD dementia, PCA, lvPPA. P-values were adjusted for multiple comparisons using the false discovery rate (FDR). Significant results indicate that the likelihood of a clinical diagnosis accurately predicting AD neuropathology varies across groups.

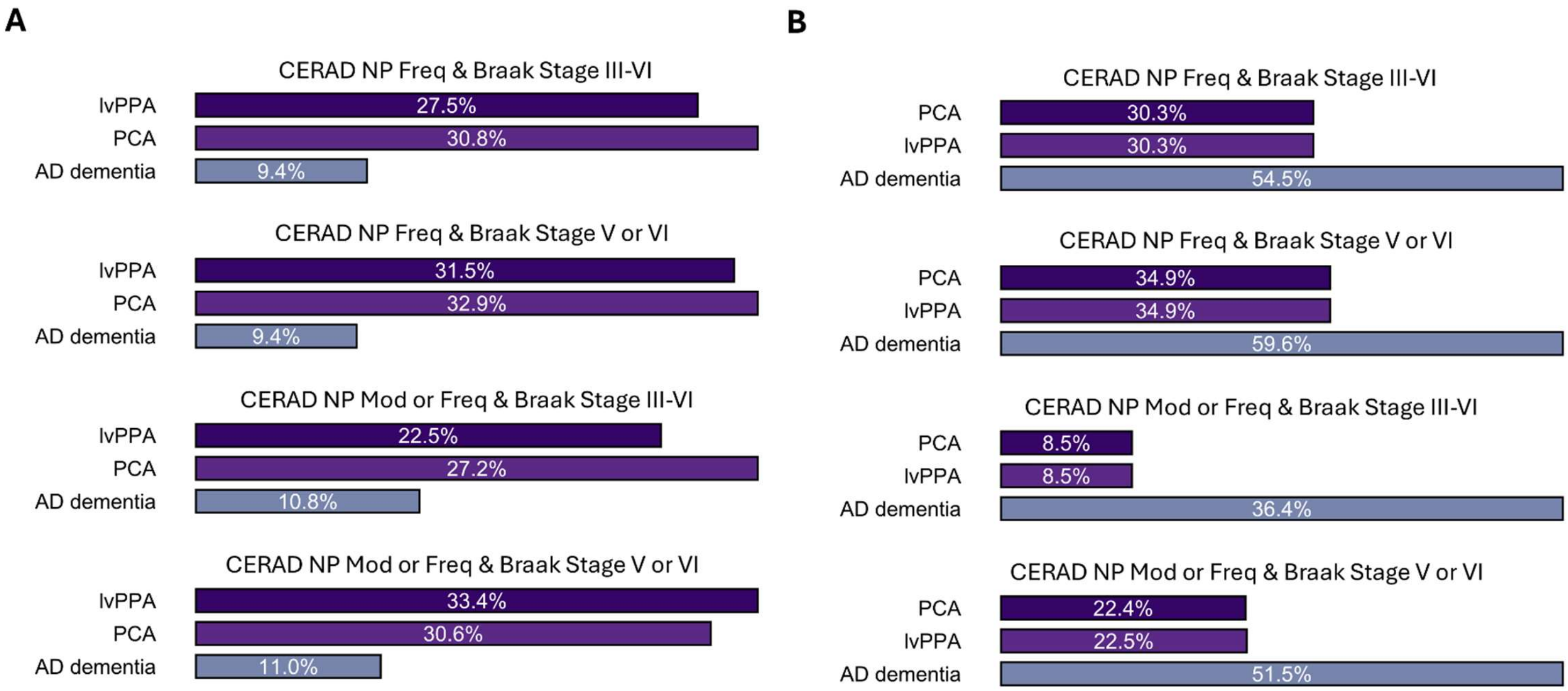

Supplementary Figure 1. (**A**) Difference between the positive predictive value (PPV) for each clinical diagnosis group and a benchmark PPV, defined as the overall PPV across all individuals (N = 6363), shown across all neuropathological thresholds. (**B**) Difference between the negative predictive value (NPV) for each clinical diagnosis group and a benchmark NPV, defined as the overall NPV across all individuals, shown across all neuropathological thresholds.

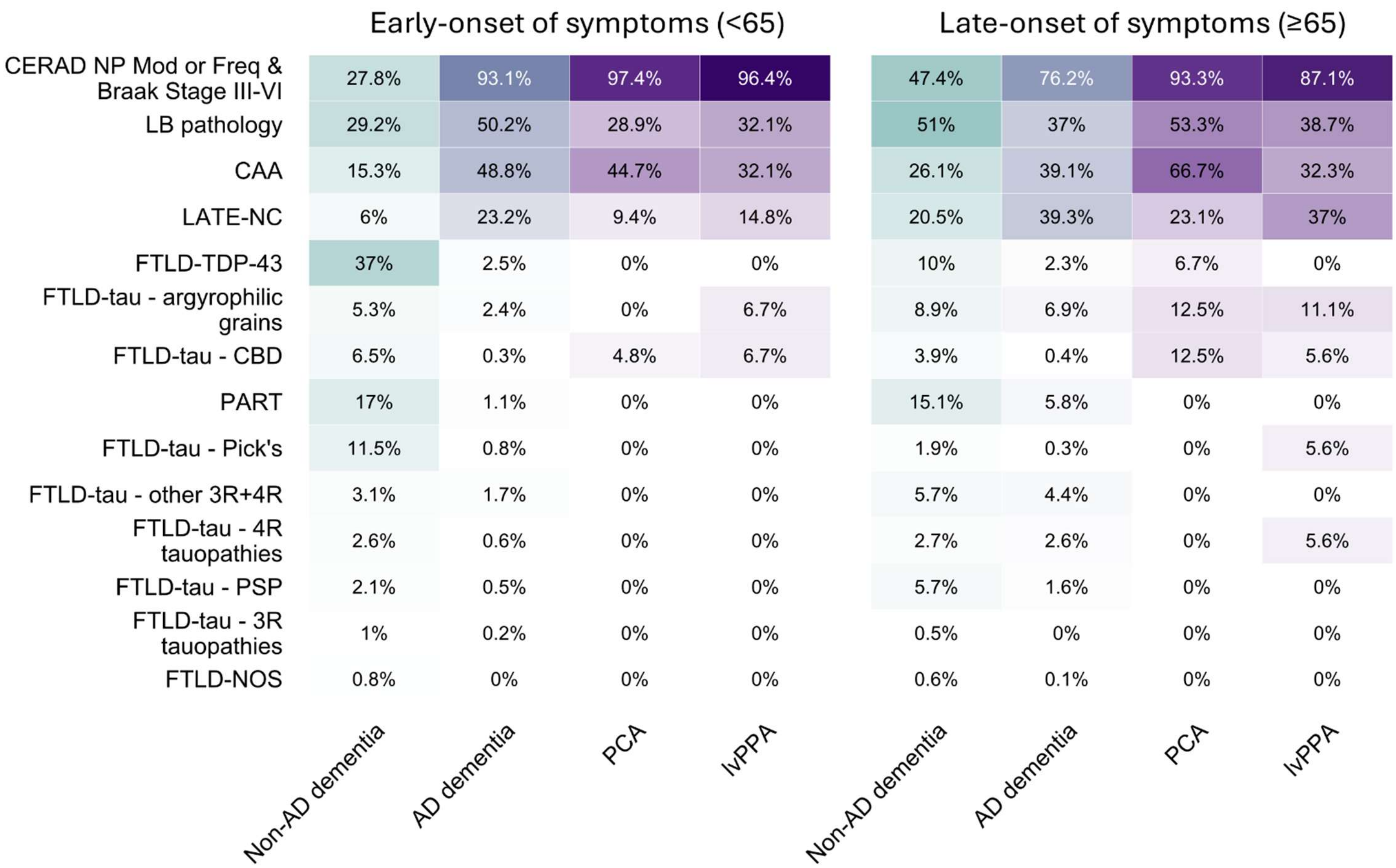

Supplementary Figure 2. Percentage of individuals (N = 6259) exhibiting different neuropathological findings in clinical groups diagnosed with non-AD dementia, amnestic AD dementia, PCA, and lvPPA, in individuals with early-onset of symptoms (left, N =1968) and late-onset of symptoms (right, N = 4291).

Pathology	Comparison	p-value	Adj. p-value (FDR)
CAA	AD dementia vs lvPPA	0.230	0.585
CAA	AD dementia vs PCA	0.159	0.571
CERAD NP Mod or Freq & Braak Stage III-VI	AD dementia vs lvPPA	0.022	0.153
CERAD NP Mod or Freq & Braak Stage III-VI	AD dementia vs PCA	0.001	**0.041**
FTLD-NOS	AD dementia vs lvPPA	1.000	1.000
FTLD-NOS	AD dementia vs PCA	1.000	1.000
FTLD-tau - 3R tauopathies	AD dementia vs lvPPA	1.000	1.000
FTLD-tau - 3R tauopathies	AD dementia vs PCA	1.000	1.000
FTLD-tau - 4R tauopathies	AD dementia vs lvPPA	0.426	0.995
FTLD-tau - 4R tauopathies	AD dementia vs PCA	1.000	1.000
FTLD-tau - argyrophilic grains	AD dementia vs lvPPA	0.204	0.571
FTLD-tau - argyrophilic grains	AD dementia vs PCA	1.000	1.000
FTLD-tau - CBD	AD dementia vs lvPPA	0.017	0.153
FTLD-tau - CBD	AD dementia vs PCA	0.014	0.153
FTLD-tau - other 3R + 4R tauopathies	AD dementia vs lvPPA	1.000	1.000
FTLD-tau - other 3R + 4R tauopathies	AD dementia vs PCA	1.000	1.000
FTLD-tau - Pick's	AD dementia vs lvPPA	0.197	0.571
FTLD-tau - Pick's	AD dementia vs PCA	1.000	1.000
FTLD-tau - PSP	AD dementia vs lvPPA	1.000	1.000
FTLD-tau - PSP	AD dementia vs PCA	1.000	1.000
FTLD-TDP-43	AD dementia vs lvPPA	1.000	1.000
FTLD-TDP-43	AD dementia vs PCA	0.473	1.000
LATE-NC	AD dementia vs lvPPA	0.058	0.326
LATE-NC	AD dementia vs PCA	0.693	1.000
LB pathology	AD dementia vs lvPPA	0.593	1.000
LB pathology	AD dementia vs PCA	0.672	1.000
PART	AD dementia vs lvPPA	0.115	0.538
PART	AD dementia vs PCA	0.180	0.571

Supplementary Table 5. Fisher’s exact tests comparing the prevalence of co-pathologies across clinical groups. This table reports uncorrected and adjusted p-values obtained using Fisher’s exact test for proportions, with false discovery rate (FDR) correction was applied to account for multiple comparison

		Early-onset		Late-onset	
Pathology	Comparison	p-value	Adj. p-value (FDR)	p-value	Adj. p-value (FDR)
AD pathology	AD dementia vs lvPPA	1	1	0.203	0.776
AD pathology	AD dementia vs PCA	0.509	1	0.218	0.776
CAA	AD dementia vs lvPPA	0.0878	0.392	0.467	0.867
CAA	AD dementia vs PCA	0.742	1	**0.0351**	0.456
LATE-NC	AD dementia vs lvPPA	0.479	1	1	1
LATE-NC	AD dementia vs PCA	0.0800	0.392	0.270	0.776
LB pathology	AD dementia vs lvPPA	0.0833	0.392	0.853	1
LB pathology	AD dementia vs PCA	0.0125	0.324	0.283	0.776
FTLD-tau - CBD	AD dementia vs lvPPA	0.0656	0.392	0.0732	0.476
FTLD-tau - CBD	AD dementia vs PCA	0.0905	0.392	**0.0333**	0.456
FTLD-tau - Pick's	AD dementia vs lvPPA	1	1	0.0611	0.476
FTLD-tau - Pick's	AD dementia vs PCA	1	1	1	1
FTLD-tau - PSP	AD dementia vs lvPPA	1	1	1	1
FTLD-tau - PSP	AD dementia vs PCA	1	1	1	1
FTLD-tau - other tauopathies	AD dementia vs lvPPA	1	1	1	1
FTLD-tau - other tauopathies	AD dementia vs PCA	1	1	1	1
FTLD-tau - argyrophilic grains	AD dementia vs lvPPA	0.325	1	0.361	0.840
FTLD-tau - argyrophilic grains	AD dementia vs PCA	1	1	0.439	0.867
FTLD-tau - 4R tauopathies	AD dementia vs lvPPA	1	1	0.388	0.840
FTLD-tau - 4R tauopathies	AD dementia vs PCA	1	1	1	1
FTLD-TDP-43	AD dementia vs lvPPA	1	1	1	1
FTLD-TDP-43	AD dementia vs PCA	1	1	0.299	0.776
PART	AD dementia vs lvPPA	1	1	0.257	0.776
PART	AD dementia vs PCA	1	1	1	1

Supplementary Table 6. Fisher's exact tests comparing the prevalence of co-pathologies across clinical groups. This table reports uncorrected and adjusted p-values obtained using Fisher's exact test for proportions, with false discovery rate (FDR) correction was applied to account for multiple comparisons

Early-onset				
**Clinical diagnosis**	**CERAD Freq & Braak III–VI**	**CERAD Freq & Braak V–VI**	**CERAD Mod or Freq & Braak III–VI**	**CERAD Mod or Freq & Braak V–VI**
AD dementia	85.9% (83.7–87.8)	84.4% (82.1–86.4)	93.1% (91.5–94.5)	90.1% (88.2–91.7)
lvPPA	85.7% (68.5–94.3)	85.7% (68.5–94.3)	96.4% (82.3–99.4)	96.4% (82.3–99.4)
Non-AD dementia	21.9% (19.2–24.9)	19.2% (16.6–22)	27.8% (24.8–31)	21.4% (18.7–24.4)
PCA	92.1% (79.2–97.3)	89.5% (75.9–95.8)	97.4% (86.5–99.5)	94.7% (82.7–98.5)
**Late-onset**				
**Clinical diagnosis**	**CERAD Freq & Braak III–VI**	**CERAD Freq & Braak V–VI**	**CERAD Mod or Freq & Braak III–VI**	**CERAD Mod or Freq & Braak V–VI**
AD dementia	54.8% (53.1–56.5)	49.9% (48.2–51.6)	76.2% (74.8–77.6)	63.2% (61.6–64.9)
lvPPA	74.2% (56.8–86.3)	74.2% (56.8–86.3)	87.1% (71.1–94.9)	87.1% (71.1–94.9)
Non-AD dementia	28.5% (25.6–31.6)	22.6% (19.9–25.5)	47.4% (44.1–50.8)	31.5% (28.5–34.7)
PCA	60% (35.7–80.2)	60% (35.7–80.2)	93.3% (70.2–98.8)	73.3% (48–89.1)

Supplementary Table 7. Positive predictive value (PPV) estimates and 95% confidence intervals across diagnoses and stratified by histopathological severity in individuals with early-onset and late-onset of symptoms.

Group	Combination	N positive	N available	Percentage
Early-onset	CAA	5	72	6.9
	LB pathology	15	75	20
	FTLD-tau - Pick's	3	69	4.3
	FTLD-tau - CBD	1	68	1.5
	FTLD-tau - PSP	1	69	1.4
	FTLD-tau - argyrophilic grains	1	34	2.9
	FTLD-tau - other tauopathies	1	34	2.9
	FTLD-TDP-43	4	35	11.4
	PART	5	75	6.7
	CAA & LB pathology	5	72	6.9
	LB pathology & FTLD-tau - Pick's	2	69	2.9
	LB pathology & FTLD-tau - other tauopathies	1	34	2.9
	LB pathology & PART	3	75	4
	FTLD-tau - PSP & PART	1	69	1.4
	FTLD-tau - 4R tauopathies & FTLD-TDP-43	1	30	3.3
	FTLD-TDP-43 & PART	2	35	5.7
	CAA & LB pathology & FTLD-TDP-43	1	35	2.9
	LB pathology & FTLD-tau - Pick's & FTLD-TDP-43	1	31	3.2
	CAA & LB pathology & FTLD-tau - argyrophilic grains & FTLD-TDP-43	1	31	3.2
	FTLD-tau - argyrophilic grains & FTLD-tau - other tauopathies & FTLD-TDP-43 & PART	1	31	3.2
Late-onset	CAA	78	792	9.8
	LB pathology	98	800	12.2
	FTLD-tau - Pick's	5	686	0.7
	FTLD-tau - CBD	1	680	0.1
	FTLD-tau - PSP	4	679	0.6
	FTLD-tau - argyrophilic grains	5	310	1.6
	FTLD-tau - 4R tauopathies	1	311	0.3
	FTLD-TDP-43	9	395	2.3
	LATE-NC	23	310	7.4
	PART	122	805	15.2
	CAA & LB pathology	42	787	5.3
	CAA & FTLD-tau - argyrophilic grains	1	310	0.3
	CAA & FTLD-tau - 4R tauopathies	1	311	0.3
	CAA & FTLD-tau - other tauopathies	2	317	0.6
	CAA & LATE-NC	7	310	2.3
	CAA & PART	8	792	1
	LB pathology & FTLD-tau - CBD	1	676	0.1
	LB pathology & FTLD-tau - PSP	2	675	0.3
	LB pathology & LATE-NC	20	310	6.5
	LB pathology & PART	28	800	3.5
	FTLD-tau - Pick's & PART	1	686	0.1
	FTLD-tau - CBD & PART	3	680	0.4
	FTLD-tau - PSP & LATE-NC	1	216	0.5
	FTLD-tau - PSP & PART	7	679	1

	FTLD-tau - argyrophilic grains & FTLD-tau - other tauopathies	1	308	0.3
	FTLD-tau - argyrophilic grains & FTLD-TDP-43	2	283	0.7
	FTLD-tau - argyrophilic grains & LATE-NC	2	210	1
	FTLD-tau - argyrophilic grains & PART	3	310	1
	FTLD-tau - 4R tauopathies & LATE-NC	3	212	1.4
	FTLD-tau - other tauopathies & FTLD-TDP-43	1	288	0.3
	FTLD-tau - other tauopathies & LATE-NC	1	216	0.5
	FTLD-TDP-43 & PART	2	395	0.5
	FTLD-NOS & LATE-NC	1	171	0.6
	LATE-NC & PART	4	310	1.3
	CAA & LB pathology & FTLD-tau - PSP	1	662	0.2
	CAA & LB pathology & FTLD-TDP-43	1	394	0.3
	CAA & LB pathology & LATE-NC	6	310	1.9
	CAA & LB pathology & PART	2	787	0.3
	CAA & FTLD-tau - argyrophilic grains & LATE-NC	2	210	1
	CAA & FTLD-tau - other tauopathies & LATE-NC	1	216	0.5
	CAA & LATE-NC & PART	1	310	0.3
	LB pathology & FTLD-tau - PSP & FTLD-TDP-43	1	288	0.3
	LB pathology & FTLD-tau - PSP & LATE-NC	2	216	0.9
	LB pathology & FTLD-tau - argyrophilic grains & FTLD-tau - other tauopathies	2	307	0.7
	LB pathology & FTLD-tau - argyrophilic grains & LATE-NC	5	210	2.4
	LB pathology & FTLD-tau - argyrophilic grains & PART	2	309	0.6
	LB pathology & FTLD-tau - 4R tauopathies & FTLD-TDP-43	1	283	0.4
	LB pathology & FTLD-tau - 4R tauopathies & LATE-NC	1	212	0.5
	LB pathology & FTLD-tau - other tauopathies & LATE-NC	1	216	0.5
	LB pathology & FTLD-TDP-43 & PART	1	394	0.3
	LB pathology & LATE-NC & PART	5	310	1.6
	FTLD-tau - CBD & FTLD-tau - argyrophilic grains & LATE-NC	1	210	0.5
	FTLD-tau - CBD & FTLD-tau - other tauopathies & LATE-NC	1	216	0.5
	FTLD-tau - PSP & FTLD-tau - argyrophilic grains & PART	1	310	0.3
	FTLD-tau - argyrophilic grains & FTLD-tau - 4R tauopathies & LATE-NC	1	206	0.5
	FTLD-tau - argyrophilic grains & FTLD-tau - other tauopathies & FTLD-TDP-43	1	282	0.4
	FTLD-tau - argyrophilic grains & FTLD-tau - other tauopathies & PART	1	308	0.3
	FTLD-tau - 4R tauopathies & FTLD-TDP-43 & PART	1	284	0.4
	CAA & LB pathology & FTLD-tau - argyrophilic grains & LATE-NC	1	210	0.5
	CAA & LB pathology & FTLD-tau - 4R tauopathies & LATE-NC	1	212	0.5
	CAA & LB pathology & FTLD-tau - other tauopathies & PART	1	316	0.3
	LB pathology & FTLD-tau - argyrophilic grains & FTLD-tau - other tauopathies & LATE-NC	2	210	1

	LB pathology & FTLD-tau - argyrophilic grains & LATE-NC & PART	1	210	0.5
	LB pathology & FTLD-tau - other tauopathies & LATE-NC & PART	1	216	0.5
	FTLD-tau - 4R tauopathies & FTLD-tau - other tauopathies & LATE-NC & PART	1	212	0.5
	CAA & LB pathology & FTLD-tau - PSP & FTLD-tau - argyrophilic grains & FTLD-TDP-43	1	282	0.4

Supplementary Table 8. Prevalence of co-pathology combinations among individuals with a false-positive clinical diagnosis of AD dementia with early- or late-onset of clinical symptoms. Each row represents a combination of co-pathologies observed in the dataset. Columns show the number of individuals positive for that combination (N positive), the number of individuals with available data for included pathologies (N available), and the percentage of individuals with that combination among those with available data (%).

Group	Pathology	N_positive	N_total	Percent
Early-onset	Vascular pathology	68	73	93.15
	LB pathology	29	75	38.67
	FTLD-TDP-43	11	35	31.43
	CAA	12	72	16.67
	PART	12	75	16.00
	FTLD-tau - other tauopathies	3	34	8.82
	FTLD-tau - argyrophilic grains	3	34	8.82
	FTLD-tau - Pick's	6	69	8.70
	FTLD-tau - 4R tauopathies	1	33	3.03
	FTLD-tau - PSP	2	69	2.90
	FTLD-tau - CBD	1	68	1.47
	LATE-NC	0	32	0.00
	FTLD-tau - 3R tauopathies	0	33	0.00
	FTLD-NOS	0	27	0.00
Late-onset	Vascular pathology	788	800	98.50
	LATE-NC	96	310	30.97
	LB pathology	230	800	28.75
	PART	196	805	24.35
	CAA	157	792	19.82
	FTLD-tau - argyrophilic grains	35	310	11.29
	FTLD-tau - other tauopathies	17	317	5.36
	FTLD-TDP-43	21	395	5.32
	FTLD-tau - 4R tauopathies	11	311	3.54
	FTLD-tau - PSP	20	679	2.95
	FTLD-tau - CBD	7	680	1.03
	FTLD-tau - Pick's	6	686	0.87
	FTLD-NOS	1	274	0.36
	FTLD-tau - 3R tauopathies	0	311	0.00

Supplementary Table 9. Marginal prevalence of pathologies among individuals with a false-positive clinical diagnosis of AD dementia with early- or late-onset of clinical symptoms.

Clinical	PPV for A+T+	NPV for A+T+
AD dementia	78.1% (61.2–89.0)	55.0% (49.2–60.8)
PCA	100.0% (87.9–100.0)	55.0% (49.1–60.7)
lvPPA	100.0% (80.6–100.0)	52.7% (47.0–58.4)

Supplementary Table 10. Positive predictive value (PPV) and negative predictive value (NPV) estimates and 95% confidence intervals across diagnoses and stratified by histopathological severity.
